# Comparative protein analysis of two maize genotypes with contrasting tolerance to low temperature

**DOI:** 10.1186/s12870-023-04198-8

**Published:** 2023-04-05

**Authors:** Salika Ramazan, Nelofer Jan, Riffat John

**Affiliations:** Plant Molecular Biology Lab, Department of Botany, University of Kashmir, Srinagar, Kashmir 190 006 India

**Keywords:** 2D-PAGE, Maize, Low temperature, Protein abundance, Stress

## Abstract

**Background:**

Low temperature (LT) stress is one of the major environmental stress factors affecting the growth and yield of maize (*Zea mays* L.). Hence, it is important to unravel the molecular mechanisms behind LT stress tolerance to improve molecular breeding in LT tolerant genotypes. In the present study, two maize genotypes viz. Gurez local from Kashmir Himalaya and tropical grown GM6, were dissected for their LT stress response in terms of accumulation of differentially regulated proteins (DRPs). Leaf proteome analysis at three-leaf stage of maize seedlings subjected to LT stress of 6 °C for a total of 12 h duration was performed using two dimensional gel electrophoresis (2D-PAGE) followed by subsequent identification of the proteins involved.

**Results:**

After MALDI-TOF (Matrix-assisted laser desorption/ionization-time of flight) and bioinformatics analysis, 19 proteins were successfully identified in Gurez local, while as 10 proteins were found to get successful identification in GM6. The interesting observations from the present investigation is the identification of three novel proteins viz. threonine dehydratase biosynthetic chloroplastic, thylakoidal processing peptidase 1 chloroplastic, and nodulin-like protein, whose role in abiotic stress tolerance, in general, and LT stress, in particular, has not been reported so far. It is important to highlight here that most of LT responsive proteins including the three novel proteins were identified from Gurez local only, owing to its exceptional LT tolerance. From the protein profiles, obtained in both genotypes immediately after LT stress perception, it was inferred that stress responsive protein accumulation and their expression fashion help the Gurez local in seedling establishment and withstand unfavorable conditions as compared to GM6. This was inferred from the findings of pathway enrichment analysis like regulation of seed growth, timing of floral transition, lipid glycosylation, and aspartate family amino acid catabolic processes, besides other key stress defense mechanisms. However, in GM6, metabolic pathways enriched were found to be involved in more general processes including cell cycle DNA replication and regulation of phenylpropanoid metabolism. Furthermore, majority of the qRT-PCR results of the selected proteins demonstrated positive correlation between protein levels and transcript abundance, thereby strengthening our findings.

**Conclusions:**

In conclusion, our findings reported majority of the identified proteins in Gurez local exhibiting up-regulated pattern under LT stress as compared to GM6. Furthermore, three novel proteins induced by LT stress were found in Gurez local, requiring further functional validation. Therefore, our results offer more insights for elucidating the molecular networks mediating LT stress tolerance in maize.

**Supplementary Information:**

The online version contains supplementary material available at 10.1186/s12870-023-04198-8.

## Background

Low temperature (LT) tolerance is a principal agronomic attribute of temperate grown crops. As changing climatic conditions threaten the global food security [1], therefore, screening out LT tolerant crops is critical for adapting climate smart agricultural practices [2]. Maize (*Zea mays* L.), a critically important food resource, and tropical in origin, is inherently susceptible to low temperatures. At any crucial stage in its life cycle, a suboptimal temperature for longer duration can cause remarkable decline in the overall growth and crop yield [3]. Ironically, the present agricultural output trend of leading food crops including maize are not adequate to meet the future requirements [4]. Hence, LT tolerance of maize must be improved by developing and selecting LT tolerant genotypes employing various traditional breeding protocols and high-throughput genomic approaches [2].

For many years now, the biological mechanisms behind LT-susceptibility of maize have been analyzed [5]. From the very first physio-morphological studies, including our previous investigations, the research revealed the impact of LT stress mostly on photosynthetic apparatus, transport processes, water relations, and plant stature [6, 7]. Additionally, the metabolomic studies have also shown that both primary and specialized metabolites accumulate within the crop in response to LT stress, which include diverse compatible solutes, antioxidant compounds, and biomass precursors [8, 9]. The availability of the complete maize genome sequence has made it possible to explore the crop’s hidden genetic potential using a variety of molecular tools and techniques [3]. It is evident from various transcriptomic studies based on the response of maize to LT stress [10, 11]. These investigations unveiled several genes coding for various transcription factors, DNA/RNA binding proteins, chromatin condensation, chloroplast functioning, circadian rhythm, and phytohormone signaling among other attributes in maize [10].

In addition to above, proteomics is a potent tool furnishing the summary of different cellular and molecular changes occurring in plants under stress conditions [12]. With respect to maize, impressive progress in the field of sequencing technologies have led to a large and fast increasing proteomic datasets (i.e., amino acid sequences and protein interaction networks). A powerful insight of these protein sequences finds many applications, such as environmental stress resistance, yield and grain quality improvement, and so on [13]. One of the versatile proteomic techniques is two-dimensional polyacrylamide gel electrophoresis (2D-PAGE) which has been widely used in differential proteomic analyses of wheat [14] and rice [15] under stress induced conditions. In case of maize, 2D-PAGE has been employed: to establish the proteome map of endosperm [16]; to analyze biotic and abiotic stress induced protein changes [17, 18]; and to study the proteomic differences in transgenic and non-transgenic maize [19].

In the above context, the present study was carried out in two maize genotypes viz. LT tolerant Gurez local and LT susceptible Gujarat-Maize-6 (G-M-6), differing in their geographical origins (temperate and tropical, respectively). The genotypes were selected to elucidate the comparative protein framework underlying the LT stress tolerance in maize seedling leaf cells, that too in very first hours of stress perception. Owing to our previous experiments on same genotypes [7, 9], interestingly, the temperate grown ‘Gurez local’ was found to exhibit exceptional LT tolerance providing a promising resource for identification of novel LT induced proteins. The proteomic (2D-PAGE and MALDI-TOF-MS) investigations revealed the altered expression of some remarkable LT responsive proteins besides identifying the novel four proteins from ‘Gurez local’. Tough subjected to further characterization and functional validation, our findings provide a comprehensive understanding of LT tolerance in maize and reveal an important role of newly identified LT regulatory proteins.

**Fig. 1:**
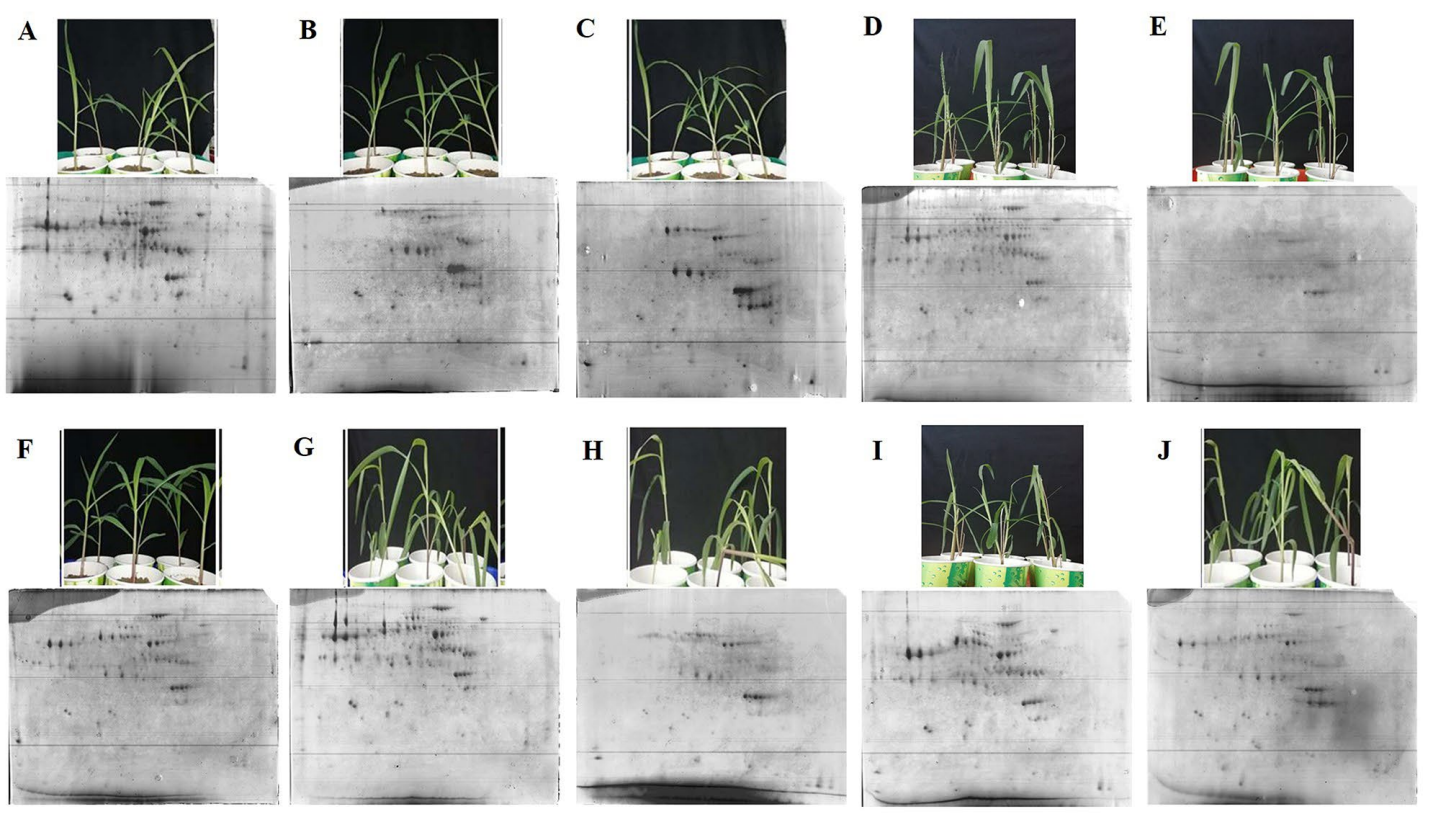
Maize seedlings **(A-E)** Gurez local **(F-J)** GM6 with their corresponding protein profiles at various LT (6 °C) stress time-points. Labels **A-E**, and **F-J** indicate the stress time points corresponding to 0 h, 2 h, 6 h, 8 and 12 h for Gurez local and GM6, respectively. The 2-DE gel is the ‘Master gel’ from the three gel replicates

## Results

### Inventory of maize seedling leaf proteins identified by 2D-PAGE

**Fig. 2:**
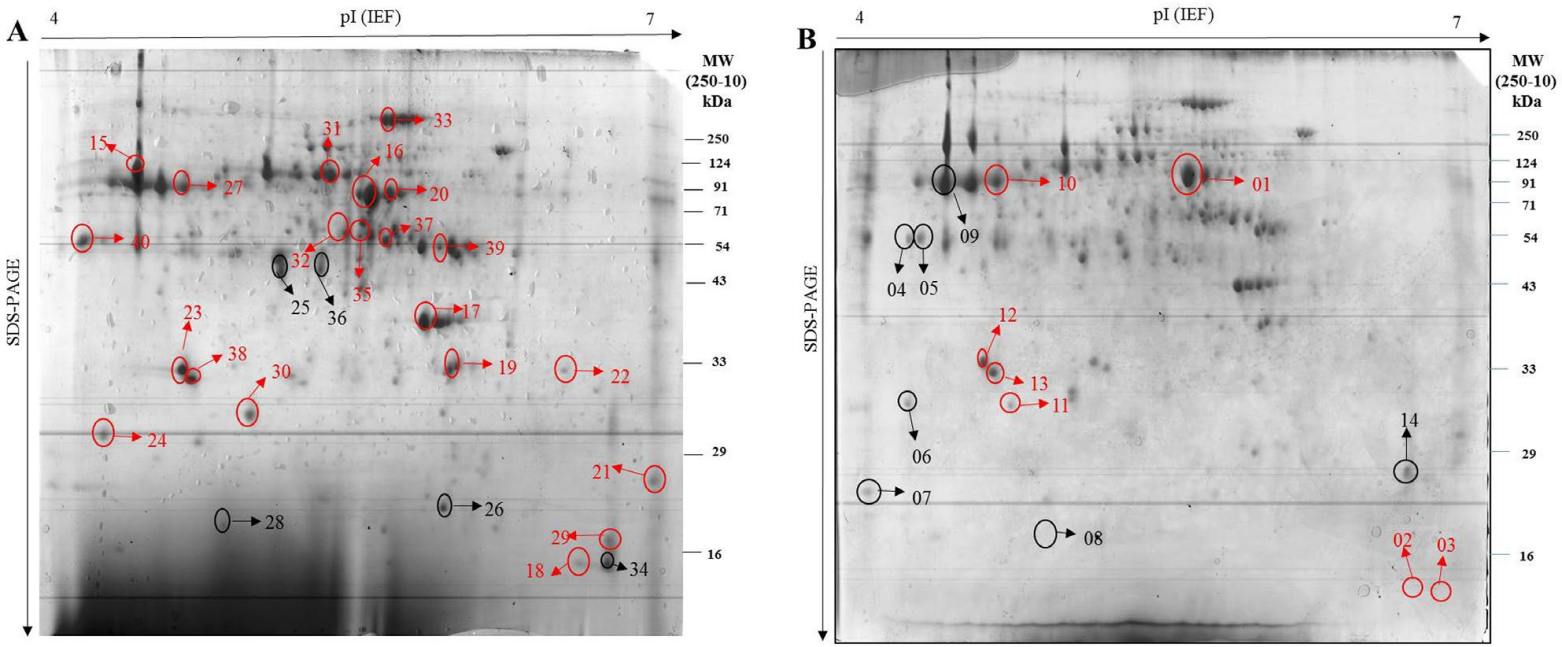
2-DE representative gel from leaves of A) Gurez local B) GM6. The numbers in the Gel images represent the protein spots with their ID’s, generated in the Image Master Gel Analysis Software. The red ellipses indicate up-regulated proteins, while the black ones indicate down-regulated proteins

**Fig. 3:**
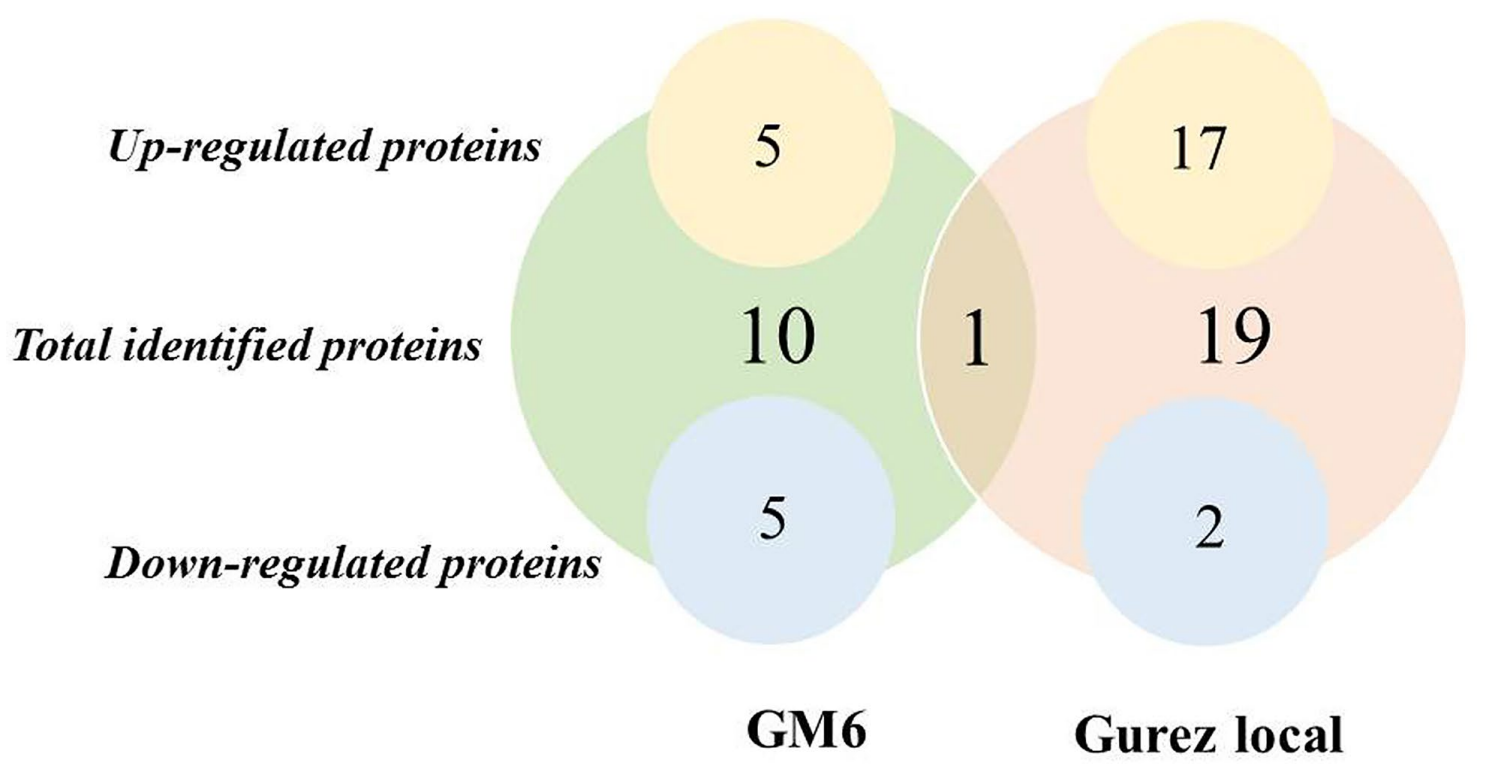
Venn diagram showing total number of identified proteins in Gurez local and GM6 through 2D-PAGE. The number in the middle of larger circles indicates total number of proteins for each genotype. The overlapping region depicts the proteins common to both genotypes induced under LT conditions. The number within smaller circles on the topside of figure represent up-regulated proteins, while the same on the downside indicate down-regulated proteins

**Fig. 4:**
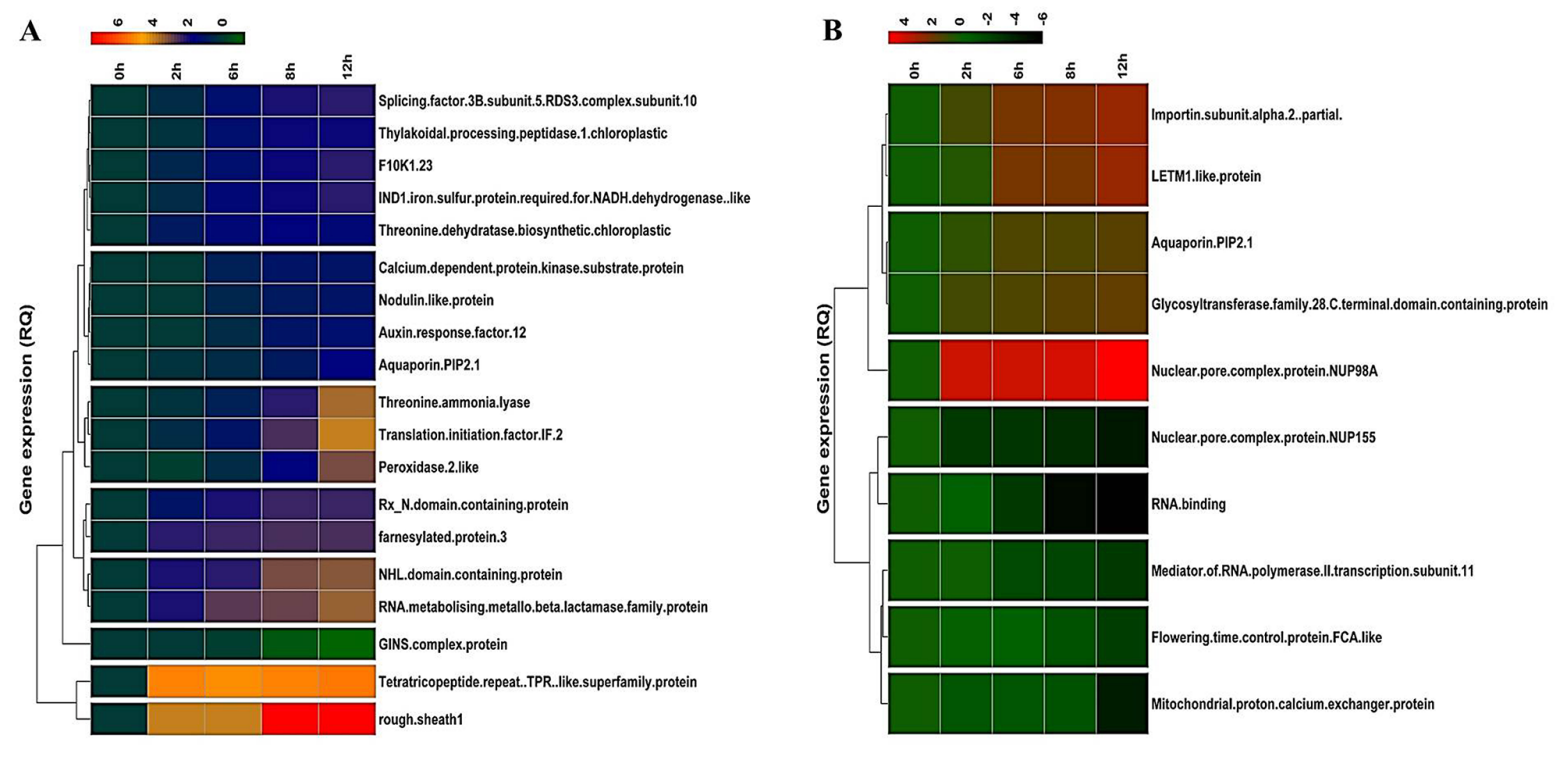
Heatmap Clustering analysis of differentially regulated proteins (DRPs) in **(A)** Gurez local **(B)** GM6. The scale bar indicates up-regulated (red) and down-regulated (blue) DRPs

**Fig. 5:**
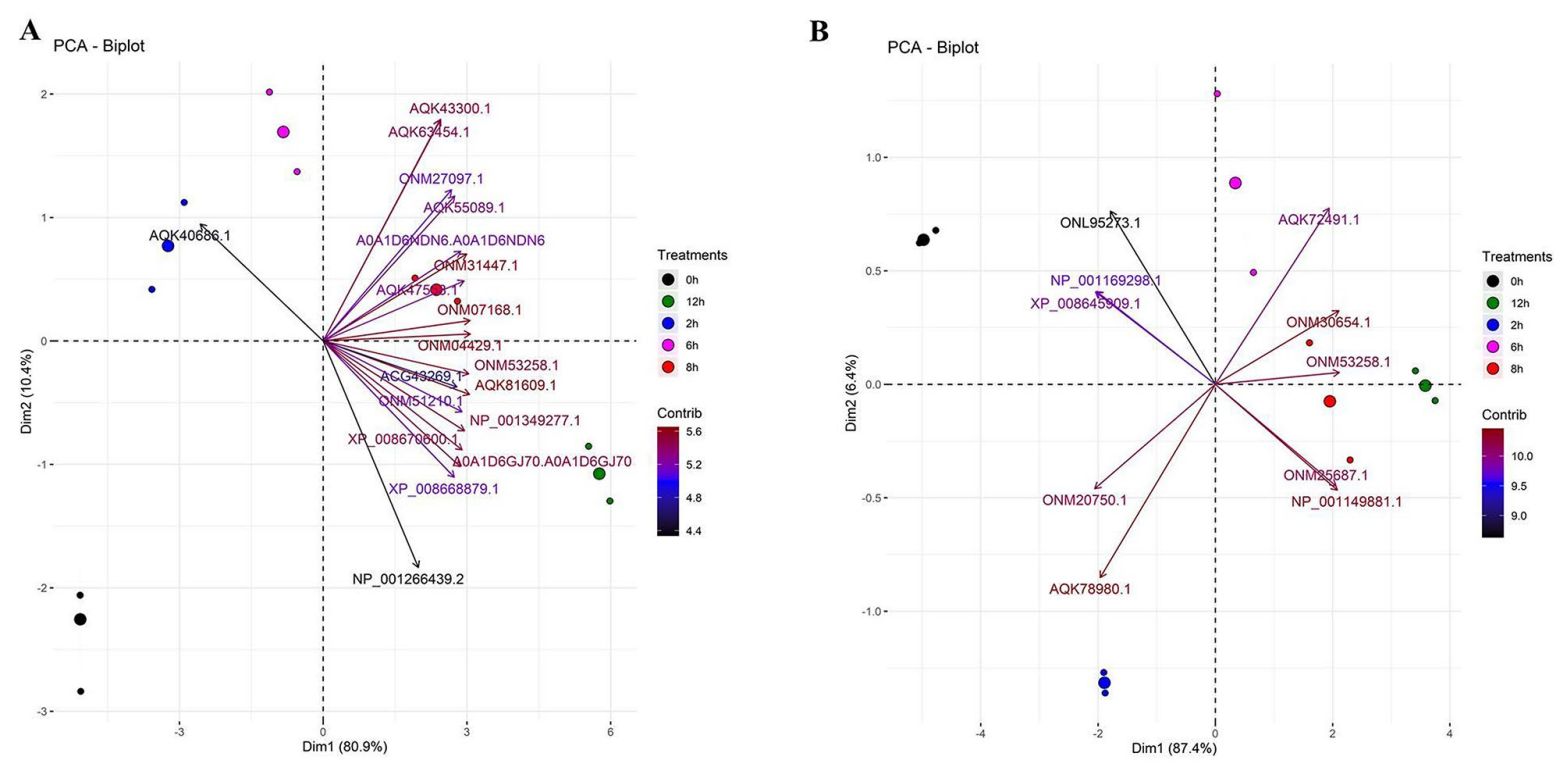
Principal Component Biplot analysis (PCA) of identified proteins with their NCBI Accession no.’s in **(A)** Gurez local, and **(B)** GM6. The scale on the right depicts contribution of each component (protein) at different LT stress time-points. Proteins with their corresponding IDs’ in Gurez local and GM6 are: Threonine dehydratase biosynthetic chloroplastic (AQK63454.1), Translation initiation factor IF-2 (XP_008668879.1), NHL domain-containing protein (AQK55089.1), Peroxidase 2-like (XP_008670600.1), Calcium-dependent protein kinase substrate protein (NP_001266439.2), Auxin response factor 12 (NP_001349277.1), Aquaporin PIP2-1 (ONM53258.1), Calcium-dependent protein kinase substrate protein (NP_001266439.2), Tetratricopeptide repeat (TPR)-like superfamily protein (ONM27097.1),, F10K1.23 (AQK81609.1), Splicing factor 3B subunit 5/RDS3 complex subunit 10 (ONM04429.1), farnesylated protein 3 (AQK43300.1), rough sheath1 (ONM51210.1), Thylakoidal processing peptidase 1 chloroplastic (AQK47553.1), GINS complex protein (AQK40686.1), Threonine dehydratase biosynthetic chloroplastic (AQK63454.1), RNA-metabolising metallo-beta-lactamase family protein (ONM31447.1) & IND1(iron-sulfur protein required for NADH dehydrogenase)-like (ONM07168.1), Threonine ammonia-lyase (A0A1D6GJ70|A0A1D6GJ70), Rx_N domain-containing protein (A0A1D6NDN6|A0A1D6NDN6) and Flowering time control protein FCA-like (NP_001169298.1), RNA binding (ONM20750.1), LETM1-like protein (AQK72491.1), Mitochondrial proton/calcium exchanger protein (XP_008645909.1), Aquaporin PIP2-1 (ONM53258.1), Mediator of RNA polymerase II transcription subunit 11 (AQK78980.1), Importin subunit alpha-2, partial (ONM30654.1), Nuclear pore complex protein NUP155 (ONL95273.1), Glycosyltransferase family 28 C-terminal domain containing protein (NP_001149881.1), Nuclear pore complex protein NUP98A (ONM25687.1), respectively

Gel analysis revealed that in Gurez local, 119 spots were obtained through database comparison and analysis, among which 80 spots were found to show up-regulated and 34 down-regulated expression. Among all these spots, only 20 spots of interest were selected based on their fold change value for MALDI-TOF analysis. Similarly, in case of GM6, total of 42 spots were found to exhibit significant changes in expression levels throughout the stress time-points, however, only 14 spots of interest based on their fold change value (p < 0.05) were selected and subjected to further analysis Fig. 2. In the end, 19 spots showed positive identification in case of Gurez local while 10 spots were identified in GM6 based on the MALDI-TOF analysis and subsequent MASCOT search as shown in Venn Diagram Fig. 3. With respect to their expression level, in Gurez local, 17 spots (pI 5–10/MW 3000-280000 Da) exhibited up regulated expression while only two were found to show down regulated expression (Spot ID 84; GINS complex protein under NCBI Accession no. AQK40686.1, and spot ID 26; F10K1.23 under NCBI Acc No. AQK81609.1). However, in GM6, 5 proteins exhibited up and 5 showed down regulated expression (pI 6–10/MW 3000–35,000 Da) (Table 1). Meanwhile, identified proteins in both genotypes under LT stress conditions revealed differences in the expression patterns as compared to control grown plants, depicted in Heatmap clustering Fig. 4. Moreover, PCA Biplot analysis also revealed that in LT tolerant Gurez local majority of the proteins contribute towards LT stress tolerance at different time intervals of the treatment as compared to control grown maize seedlings as well as in comparison to GM6 Fig. 5. It can also be assumed from PCA Biplot analysis that all these proteins get expressed on exposure to LT stress only in case of LT tolerant Gurez local as compared to GM6. Three novel proteins were isolated from the protein inventory in the Gurez local (Table 1). These proteins include Threonine dehydratase biosynthetic chloroplastic, Thylakoidal processing peptidase 1 chloroplastic, and Nodulin-like protein, whose further study may offer more information about their predicted roles in LT tolerance in maize.

**Table 1:** Differentially expressed proteins in maize genotypes in response to LT

	Spot ID	NCBI Accession Number	Protein name	pI	MW (Da)	MASCOT Score	Fold Change	Regulated Type
***Zea mays (Gurez local)***	57	ONM31447.1	RNA-metabolising metallo-beta-lactamase family protein [Zea mays]	6.03	23,819	40	10.7891	Up regulated
	167	ONM51210.1	rough sheath1 [Zea mays]	5.64	285,450	46	14.5208	Up regulated
	153	AQK63454.1	Threonine dehydratase biosynthetic chloroplastic [Zea mays]	9.68	30,407	51	41.2574	Up regulated
	18	AQK47553.1	Thylakoidal processing peptidase 1 chloroplastic [Zea mays]	10.06	20,281	37	3.54261	Up regulated
	149	AQK43300.1	farnesylated protein 3 [Zea mays]	5.25	40,895	38	4.45764	Up regulated
	165	AQK55089.1	NHL domain-containing protein [Zea mays]	6.69	44,718	40	4.11602	Up regulated
	23	ONM53258.1	Aquaporin PIP2-1 [Zea mays]	6.52	3203	23	3.0303	Up regulated
	75	NP_001349277.1	Auxin response factor 12 [Zea mays]	5.95	9099.3	48	2.32466	Up regulated
	64	ONM07168.1	IND1(iron-sulfur protein required for NADH dehydrogenase)-like [Zea mays]	10.17	10,534	31	4.20944	Up regulated
	32	ONM04429.1	Splicing factor 3B subunit 5/RDS3 complex subunit 10 [Zea mays]	6.52	3203	23	4.16235	Up regulated
	84	AQK40686.1	GINS complex protein [Zea mays]	8.64	2293.28	56	2.49025	Down regulated
	26	AQK81609.1	F10K1.23 [Zea mays]	9.86	21,278	40	4.38033	Down regulated
	52	XP_008668879.1	Translation initiation factor IF-2 [Zea mays]	10.94	3584.84	57	14.2597	Up regulated
	63	A0A1D6GJ70|A0A1D6GJ70	Threonine ammonia-lyase OS = Zea mays	5.21	32,881	36	11.5956	Up regulated
	148	A0A1D6NDN6|A0A1D6NDN6	Rx_N domain-containing protein	9.51	3102.67	49	6.37876	Up regulated
	81	ACG43269.1	Nodulin-like protein [Zea mays]	8.87	48,806	36	10.6314	Up regulated
	82	XP_008670600.1	Peroxidase 2-like [Zea mays]	5.24	29,956	42	7.93804	Up regulated
	159	ONM27097.1	Tetratricopeptide repeat (TPR)-like superfamily protein [Zea mays]	6.29	42,195	36	33.5755	Up regulated
	122	NP_001266439.2	Calcium-dependent protein kinase substrate protein [Zea mays]	9.93	16,833	37	2.0599	Up regulated
***Zea mays (GM6)***	220	AQK72491.1	LETM1-like protein [Zea mays]	9.94	4361.72	11	9.12254	Up regulated
	1964	XP_008645909.1	Mitochondrial proton/calcium exchanger protein [Zea mays]	6.05	8611.6	11	8.17725	Down regulated
	1972	NP_001169298.1	Flowering time control protein FCA-like [Zea mays]	9.08	7925.87	38	4.02113	Down regulated
	310	ONM20750.1	RNA binding [Zea mays]	9.24	25,210	38	4.22351	Down regulated
	180	AQK78980.1	Mediator of RNA polymerase II transcription subunit 11 [Zea mays]	6.70	6426	45	5.22440	Down regulated
	3349	NP_001149881.1	Glycosyltransferase family 28 C-terminal domain containing protein [Zea mays]	7.63	4766.59	50	2.94550	Up regulated
	1279	ONM25687.1	Nuclear pore complex protein NUP98A [Zea mays]	10.88	17,084	34	3.20661	Up regulated
	5275	ONM30654.1	Importin subunit alpha-2, partial [Zea mays]	10.14	35,762	51	5.98263	Up regulated
	178	ONM53258.1	Aquaporin PIP2-1 [Zea mays]	6.25	3203	23	2.08445	Up regulated
	6241	ONL95273.1	Nuclear pore complex protein NUP155 [Zea mays]	9.62	25,430	43	2.56641	Down regulated

### Generation of 3D models and their stereochemical validation

The three-dimensional models (3D) necessary for visualization and better understanding of secondary structural features of all identified proteins were successfully generated with > 80% residues modelled at ≥ 90% confidence (Fig. S1). In addition, the PDB files obtained from Phyre2 server (http://www.sbg.bio.ic.ac.uk/phyre2/html/page.cgi?id=index) uploaded at PDBsum (https://www.ebi.ac.uk/thornton-srv/databases/cgi-bin/pdbsum/GetPage.pl?pdbcode=index.html) gave us the stereochemical validation of all protein models in the form of Ramachandran Plots (RC plots) (Fig. S2). The RC plot statistics (Table S1) showing the distribution of main Ф-ψ angles in relation to ‘core’ (red) and ‘allowed’ (brown) regions, with residues falling in the ‘generously allowed’ (dark yellow) and ‘disallowed’ (pale yellow) regions plotted as red squares were successfully obtained for all the proteins.

### Classification of LT responsive proteins in both genotypes

**Fig. 6:**
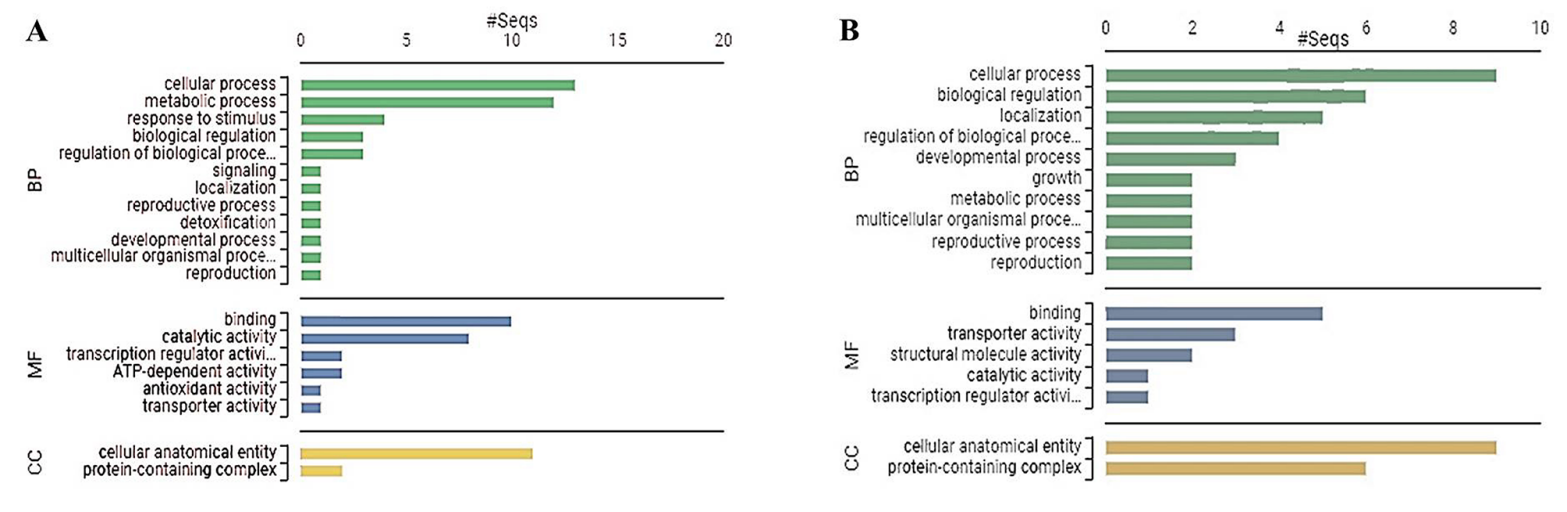
GO functional categorization based on biological process, molecular function and cellular component of identified proteins in **(A)** Gurez local **(B)** GM6 seedling leaves. The scale above represents the number of proteins contributing in each function

Online servers viz. CLAP and Blast2GO (B2G) were used to classify the identified proteins respectively based on an alignment-free local sequence similarity computing approach and the functional annotations Gene ontology functional analysis revealed that although most of the GO terms are shared between the two genotypes, but the differential regulation patterns (DRPs) vary considerably in Gurez local from that of GM6 with respect to GO distribution level in terms of metabolic processes (GO: 0008152), biological regulation (GO: 0065007), and localization (GO: 0051179). However, 3 terms were found to be unique to Gurez local including the response to stimulus (GO: 0050896), signaling (GO: 0023052), and detoxification (GO: 0098754) under ‘biological process’ category. With regards to molecular functions, ATP dependent activity (GO: 0140657) and antioxidant activity (GO: 0016209) were specifically enriched in Gurez local only while rest other terms were shared between the two genotypes (Fig. 6).

**Fig. 7:**
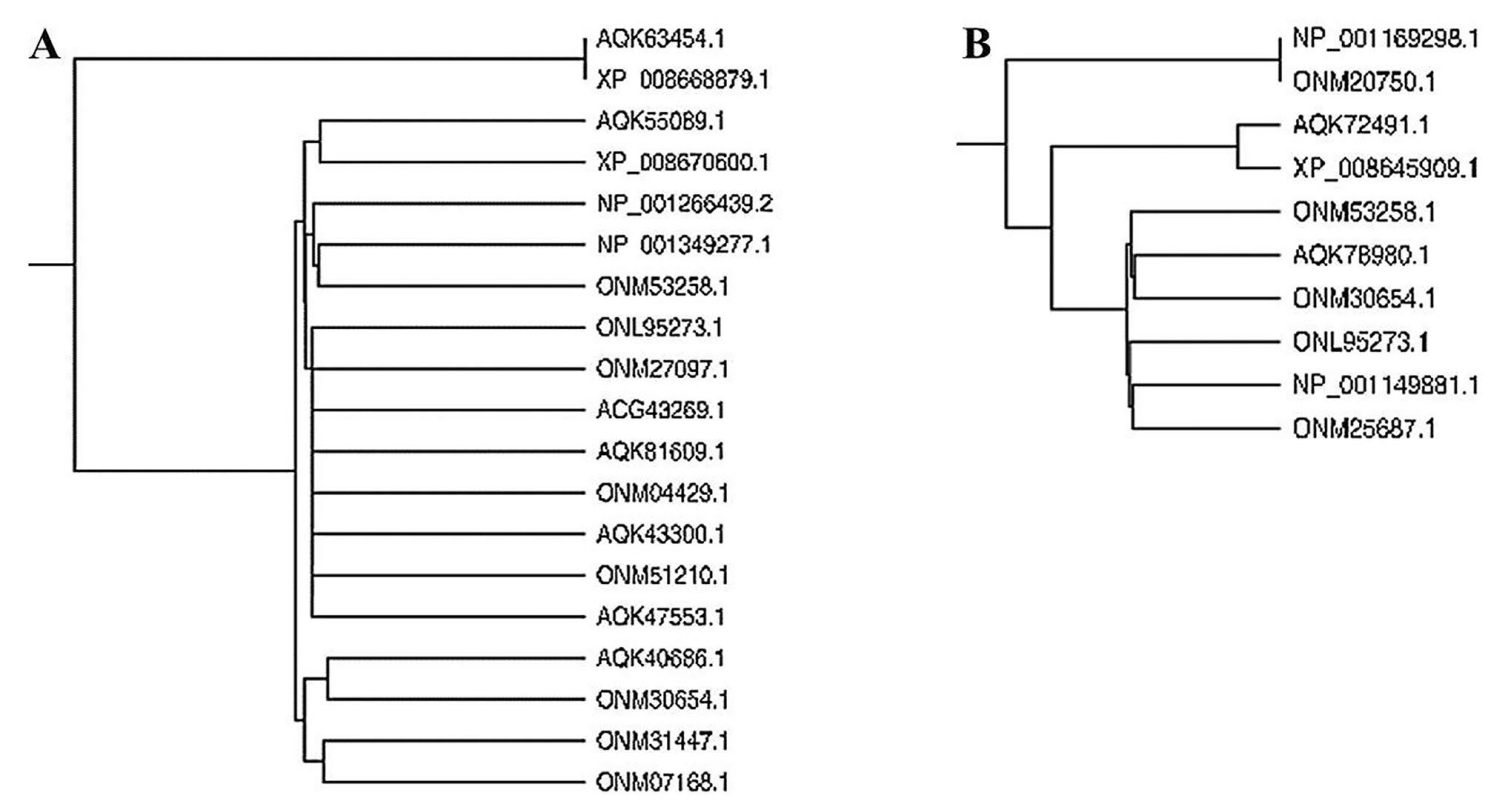
Hieracrchiel clustering exhibiting high domain architectural similarities of identified proteins in **(A)** Gurez local **(B)** GM6, protein sequences labelled as NCBI Accession numbers. In Gurez local, Threonine dehydratase biosynthetic chloroplastic (AQK63454.1) and Translation initiation factor IF-2 (XP_008668879.1) formed a biologically meaningful group of two proteins while rest 17 proteins clustered together into another group. The later was found to form 4 clusters each containing closely related set of proteins [I: NHL domain-containing protein (AQK55089.1) & Peroxidase 2-like (XP_008670600.1)]; [II: Calcium-dependent protein kinase substrate protein (NP_001266439.2), Auxin response factor 12 (NP_001349277.1), Aquaporin PIP2-1 (ONM53258.1)]; [III: Calcium-dependent protein kinase substrate protein (NP_001266439.2), Tetratricopeptide repeat (TPR)-like superfamily protein (ONM27097.1),, F10K1.23 (AQK81609.1), Splicing factor 3B subunit 5/RDS3 complex subunit 10 (ONM04429.1), farnesylated protein 3 (AQK43300.1), rough sheath1 (ONM51210.1) & Thylakoidal processing peptidase 1 chloroplastic (AQK47553.1)] and [IV: GINS complex protein (AQK40686.1), Threonine dehydratase biosynthetic chloroplastic (AQK63454.1), RNA-metabolising metallo-beta-lactamase family protein (ONM31447.1) & IND1(iron-sulfur protein required for NADH dehydrogenase)-like (ONM07168.1)] separated at distinct nodes. In GM6, 3 groups of proteins were obtained. In the first two groups, 2 proteins each [(Flowering time control protein FCA-like (NP_001169298.1), RNA binding (ONM20750.1) & LETM1-like protein (AQK72491.1), Mitochondrial proton/calcium exchanger protein (XP_008645909.1)] were clustered together, however, the third group splitted further into another two distinct clusters of three proteins each [Aquaporin PIP2-1 (ONM53258.1), Mediator of RNA polymerase II transcription subunit 11 (AQK78980.1), Importin subunit alpha-2, partial (ONM30654.1) & Nuclear pore complex protein NUP155 (ONL95273.1), Glycosyltransferase family 28 C-terminal domain containing protein (NP_001149881.1), Nuclear pore complex protein NUP98A (ONM25687.1) based on the set of closely connected proteins

Furthermore, in CLAP (Classification of Proteins), results in the form of Hieracrchiel clustering for both genotypes the protein sequences labelled as ‘NCBI Accession numbers’ were efficiently clustered into groups exhibiting high domain architectural similarities (Fig. 7). In Gurez local, proteins viz. Threonine dehydratase biosynthetic chloroplastic (Spot ID 153; NCBI Acc No. AQK63454.1) and Translation initiation factor IF-2 (Spot ID 52; NCBI Acc No. XP_008668879.1) formed a biologically meaningful group of two proteins while rest 17 proteins clustered together into another group. The later was found to form 4 clusters each containing closely related set of proteins separated at distinct nodes.

Similarly, in GM6 for clustering illustrations, 3 groups of proteins were obtained. In the first two groups, 2 proteins were clustered together, however, the third group splitted further into another two distinct clusters based on the set of closely connected proteins.

### Enriched metabolic pathways of differentially regulated proteins (DRPs) in Gurez local and GM6

**Fig. 8:**
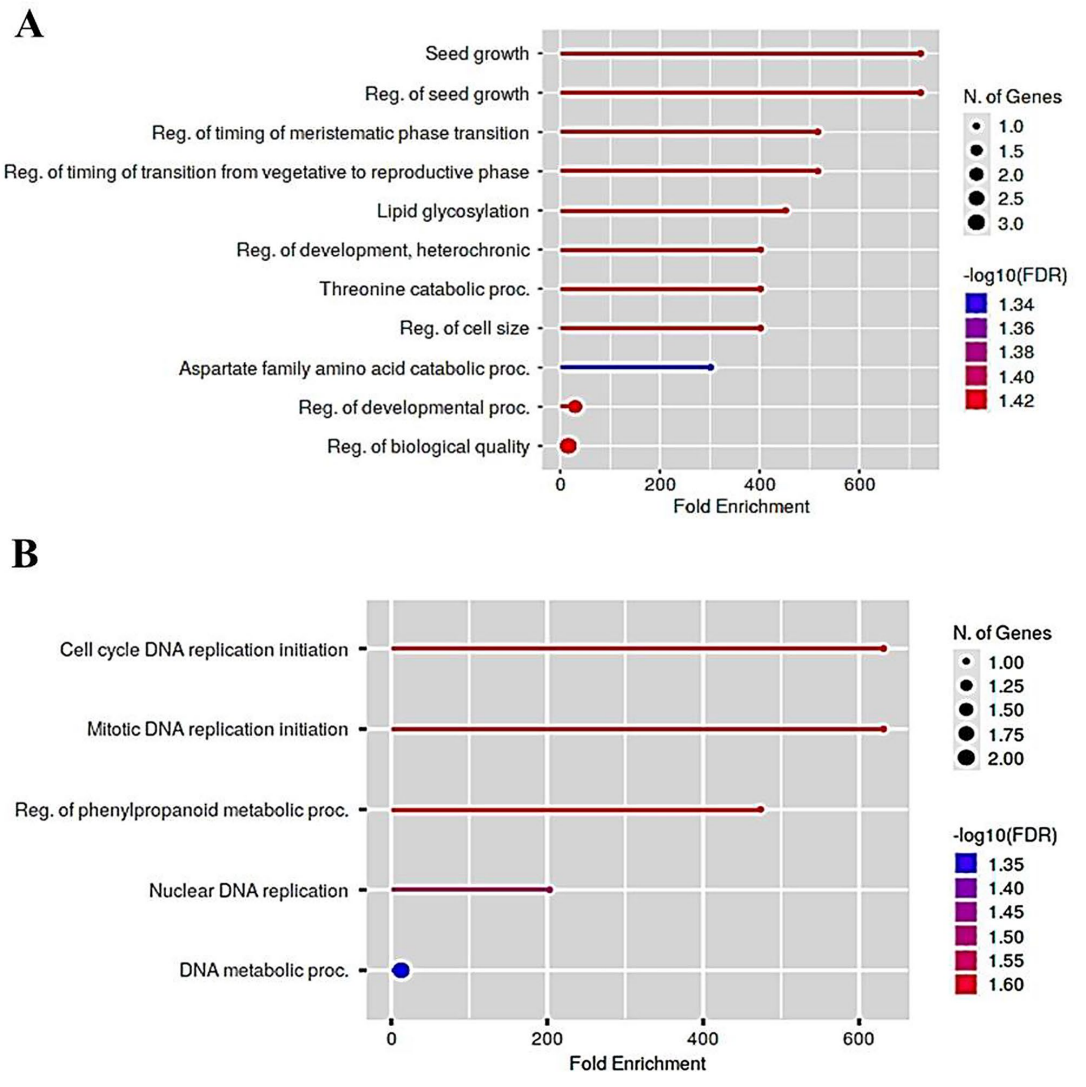
KEGG pathway enrichment analysis in DRPs in **(A)** Gurez local **(B)** GM6. The size of point represents the number of proteins enriched in a particular pathway and the X-axis represents fold enrichment value. KEGG, as developed by Kanehisa Laboratories (10.1093/nar/28.1.27), was used in the imagery

To further analyze the functional fates of DRPs, KEGG enrichment analysis using ShinyGO (http://bioinformatics.sdstate.edu/go/) was performed. It was observed that higher protein numbers were constituted in enriched pathways in case of Gurez local as compared to the GM6 (Fig. 8), Also, the two genotypes were found to exhibit a remarkable divergence in metabolic pathway responses to LT stress. Using hyper geometric test, metabolic pathways with –log10 (FDR) value greater than 1.2 were considered to be significantly affected by LT stress. We observed that in case of LT tolerant genotype Gurez local, regulation of seed growth, regulation of timing of transition from vegetative to reproductive phase, lipid glycosylation and aspartate family amino acid catabolic processes were significantly enriched (Fig. S3). Contrastingly, in GM6, cell cycle DNA replication initiation, and regulation of phenylpropanoid metabolic processes were remarkably enriched. Apart from two enriched metabolic processes (aspartate family amino acid catabolic processes and threonine catabolic processes) in the case of Gurez local (Fig. S4), most metabolic processes also clustered together in a network showing an interactive fashion of metabolic pathways. However, GM6 displayed a single cluster network of enriched pathways apart from the regulation of phenylpropanoid metabolic process (Fig. S5). Though additional studies are required for empirical observations of the metabolic events, and the growth and developmental transitions in plants (including maize) where the identified proteins apparently play a role, following are some of the enriched pathways which are pertinent to mention in Gurez local:

#### Regulation of seed growth

The leaf proteome revealed that under LT stress conditions, for regulation of seed growth, a number of proteins change their expression fashion. These include upregulated proteins viz. NHL domain-containing protein (Spot ID 165: NCBI Acc No. AQK55089.1) modulating the seed germination under abiotic stress, RNA-metabolising metallo-beta-lactamase family protein (Spot ID 57: NCBI Acc. No. ONM31447.1) involved in DNA/RNA metabolism, and downregulated proteins, such as, GINS complex (Spot ID 84: NCBI Acc No. AQK40686.1) protein and F10K1.23 (Spot ID 26: NCBI Acc No. AQK81609.1) playing a critical role in chromosomal DNA replication. All these proteins regulate the seed growth and aid in seedling emergence and its establishment.

#### Regulation of timing of transition from vegetative to reproductive phase

Developmental transitions from embryonic to post embryonic mode (seed germination), and vegetative to flowering phase, are regulated by the environmental cues [27]. The transition is also controlled by a complex genetic network including miRNAs, various transcriptional factors, and altered protein accumulation [28]. In the present study, proteins found in higher abundance under LT treatment include: ‘Thylakoidal processing peptidase 1 chloroplastic’ (Spot ID 18: NCBI Acc No. AQK47553.1) required for choloroplast protein degradation/chloroplast development/proper thylakoidal development in photosynthetic tissues; ‘rough sheath1’ (Spot ID 167: NCBI Acc No. ONM51210.1) necessary for cell fate/cell division; ‘translation initiation factor IF-2’ (Spot ID 52: NCBI Acc No. XP_008668879.1) controlling different translational processes during vegetative and reproductive growth; ‘calcium-dependent protein kinase substrate protein’ (Spot ID 122: NCBI Acc No. NP_001266439.2) critical for flowering; and ‘splicing factor 3B subunit 5/RDS3 complex subunit 10’ (Spot ID 32: NCBI Acc No. ONM04429.1) for checking the growth of terminal buds. All the above mentioned proteins accumulate in higher proportions in stress response in Gurez local and play a critical roles in floral transitions.

#### Lipid glycosylation

Glycosylation is one of the fundamental post-translational modifications modulating various developmental processes including stress response in plants [29]. Remarkably, under LT stress conditions, glycerolipids serve as signaling molecules and also protect organeller membranes from stress induced damage [30]. Interestingly, one of the novel proteins found in Gurez local viz. Nodulin-like protein is believed to have a probable role in transport of various carbohydrate meoties or other solutes that may be indirectly involved in lipid glycosylation process under LT stress conditions, though subject to further investigations.

#### Aspartate family amino acid catabolic processes and threonine catabolic processes

The co-regulation activity of aspartate coupled with other amino acids belonging to ‘Asp family amino acids’ is beneficial for plant stress adaptation [31]. Hence, the up-regulation of Threonine dehydratase biosynthetic chloroplastic (Spot ID 153: NCBI Acc No. AQK63454.1) and Threonine ammonia-lyase (Spot ID 63: Uniprot ID. A0A1D6GJ70) proteins in Gurez local reveal their potent roles in amino acid metabolism associated LT stress tolerance in maize.

#### Other pathways involved in stress response in Gurez local

Under environmental stress induced conditions, multiple signaling pathways converge to regulate stress induced genes that in turn produce proteins and enzymes required for stress metabolism in maize [32]. The present study revealed that most of the proteins accumulated in higher levels under LT stress in Gurez local are involved in defense mechanism against LT stress. The accumulation of various abiotic stress responsive proteins include farnesylated protein (Spot ID 149: NCBI Acc. No. AQK43300.1), auxin response factor 12 (Spot ID 75: NCBI Acc. No. NP_001349277.1), IND1 (iron-sulfur protein required for NADH dehydrogenase)-like (Spot ID 64: NCBI Acc. No. ONM07168.1), translation initiation factor IF-2 (Spot ID 52: NCBI Acc. No. XP_008668879.1), and tetratricopeptide repeat (TPR)-like superfamily protein (Spot ID 159: NCBI Acc. No. ONM27097.1), acting as a negative regulator in cold stress signaling. Other proteins exhibiting upregulated expression and having an indispensable role in both abiotic and biotic stress tolerance constitute NHL domain-containing protein (Spot ID 165: NCBI Acc. No. AQK55089.1), aquaporin PIP2-1 (Spot ID 23: NCBI Acc. No. ONM53258.1) and peroxidase 2-like (Spot ID 82: NCBI Acc. No.XP_008670600.1). However, another protein viz. Rx_N domain-containing protein (Spot ID 148: NCBI Acc. No. A0A1D6NDN6) which gets remarkably induced under LT treatment in Gurez local maize is well-known for its critical role in biotic stress tolerance in plants [33].

### Enriched metabolic processes in GM6 as follows

#### Cell cycle DNA replication initiation, RNA metabolism and nucleo-cytoplasmic trafficking

Protein activity generated after mRNA translation and other post-transcriptional/translational modifications involving a plethora of molecular machinery, directly or indirectly contribute to adaptation at cellular level under stress induced conditions [34]. As evident from the protein list obtained in the study (Table 1), few proteins playing a vital role in nuclear metabolism which include ‘RNA binding protein’ (Spot ID 310: NCBI Acc No. ONM20750.1) and Mediator of RNA polymerase II transcription subunit 11 (Spot ID 180: NCBI Acc. No. AQK78980.1) display downregulated expression in LT sensitive genotype. Contrastingly, the other two important nuclear proteins responsible for nuclear transport i.e., nuclear pore complex protein NUP98A (Spot ID: 1279: NCBI Acc. No. ONM25687.1) and importin subunit alpha-2, partial (Spot ID: 5275: NCBI Acc. No. ONM30654.1) accumulate in higher proportion in GM6 in response to the LT stress.

#### Other stress responsive pathways

Numerous stress factors affect flowering time in plants [35], transmembrane water movement [36] and exchange of other small molecules across mitochondria [37]. The proteins found in significant abundance in above mentioned stress signaling pathways in GM6 include aquaporin PIP2-1 (Spot ID 178: NCBI Acc. No. ONM53258.1) and LETM1-like protein (Spot ID 220: NCBI Acc. No. AQK72491.1). On the other hand, flowering time control protein FCA-like (Spot ID 1972: NCBI Acc. No. NP_001169298.1) and mitochondrial proton/calcium exchanger protein (Spot ID: 1964: NCBI Acc. No. XP_008645909.1) were found in lower abundance with LT treatment progression in GM6. Other metabolic processes involving transfer of sugar moieties onto a variety of small molecules involve the glycosyltransferase family 28 C-terminal domain containing protein (Spot ID 3349: NCBI Acc. No. NP_001149881.1) which exhibited relatively higher levels in LT sensitive genotype.

### Protein-protein interaction networks are associated with LT stress response

**Fig. 9:**
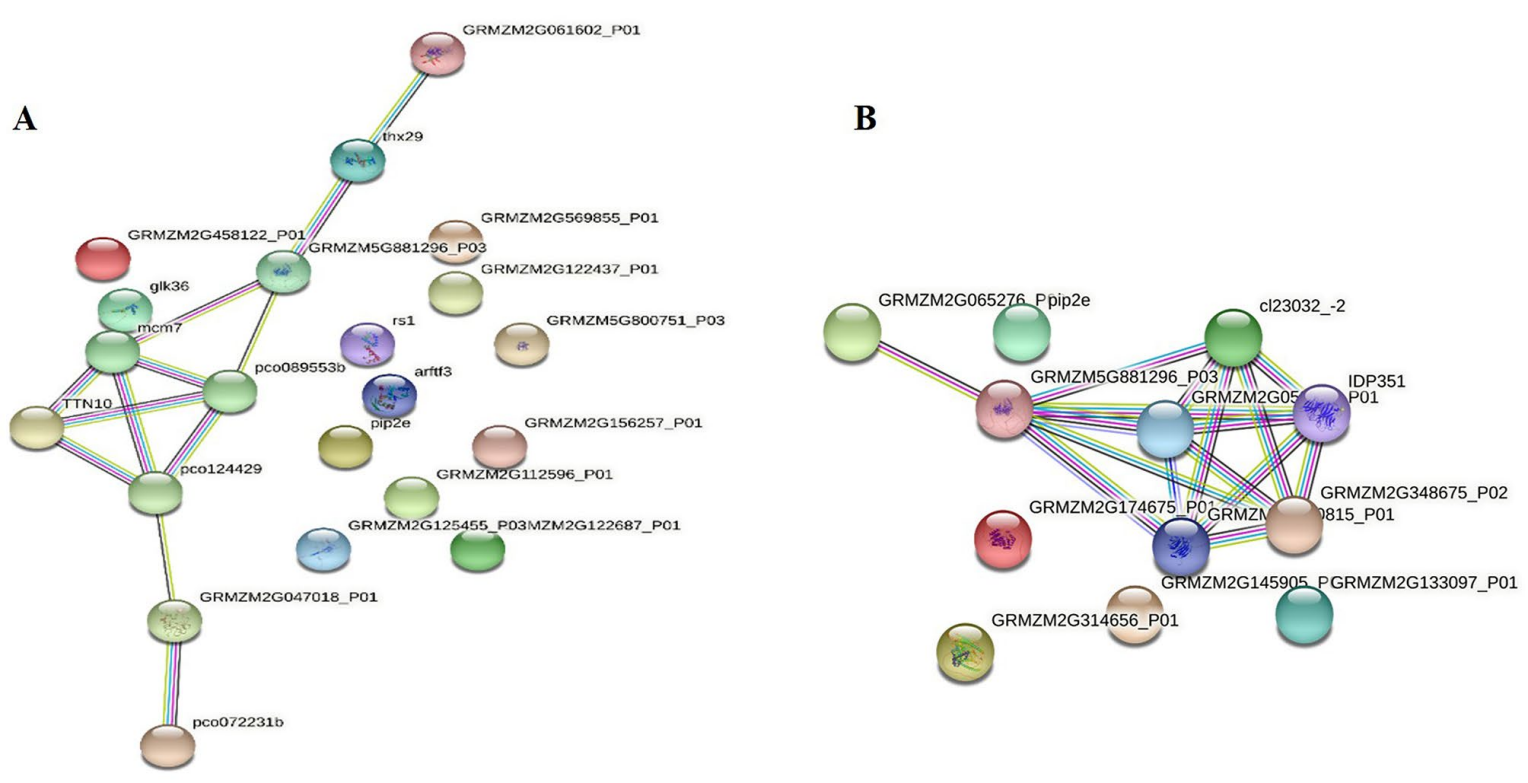
Protein interaction networks of identified proteins in **(A)** Gurez local **(B)** GM6. The network was generated with the help of STRING (https://string-db.org/) program at a confidence score greater than 0.7. Nodes (colored circles) indicate proteins and the thickness of lines connecting the nodes denotes the strength of supplementary data. Different types of interactions between nodes are represented by colored lines. Red lines indicate the fusion of genes, green lines neighborhood of genes, blue lines co-occurrence across species, purple lines experimental evidence, yellow lines text mining of abstracts from literature, light blue lines databases, and black lines co-expression in the same of other species

Using STRING 10.5 database (http://www.string-db.org/, accessed on 18 April 2022) protein-protein interaction and functional relationships among the differentially regulated proteins was predicted with the confidence score greater than 0.7 in both genotypes of maize. In case of Gurez local, single group constituting 9 interacting proteins was identified in the network. These include splicing factor 3B subunit 5/RDS3 complex subunit 10 (GRMZM2G121942_P02, pco072231b), GINS complex protein (GRMZM2G049536_P02, pco124429), NHL domain-containing protein (GRMZM2G326783_P01, thx29), DNA helicase protein (GRMZM2G139894_P01, mcm7), GINS complex protein with a predicted functional partner ‘Flowering time control protein FY’ (GRMZM5G881296_P03), GINS complex protein (GRMZM2G076128_P01, TTN10), Protein spa-1 related 4 isoform X1(GRMZM2G061602_P01), DNA replication complex protein (GRMZM2G134295_P03, pco089553b), and PHD finger-like domain containing protein 5 A (GRMZM2G047018_P01) (Fig. 9). All these proteins take part in chromosomal DNA replication.

On the other side, GM6 also exhibited a single cluster of 7 proteins in the interaction network. These include mediator of RNA polymerase II transcription subunit 11 (GRMZM2G180815_P01), nuclear pore complex protein NUP98A (GRMZM2G348675_P02), nuclear pore complex protein NUP155 (GRMZM2G057853_P01, IDP351), importin subunit alpha-2, partial (GRMZM2G059015_P01, cl23032_-2), component of nuclear pore complex (GRMZM2G058498_P01), and RNA binding protein (GRMZM5G881296_P03). All these proteins together aid in nucleocytoplasmic transport of proteins and RNAs (Fig. 9). These results indicate that interaction among various proteins especially involved in a particular metabolic pathway is essential in responding to the low temperature stress in maize.

### Expression levels of genes encoding DRPs in response to LT in maize genotypes

**Fig. 10:**
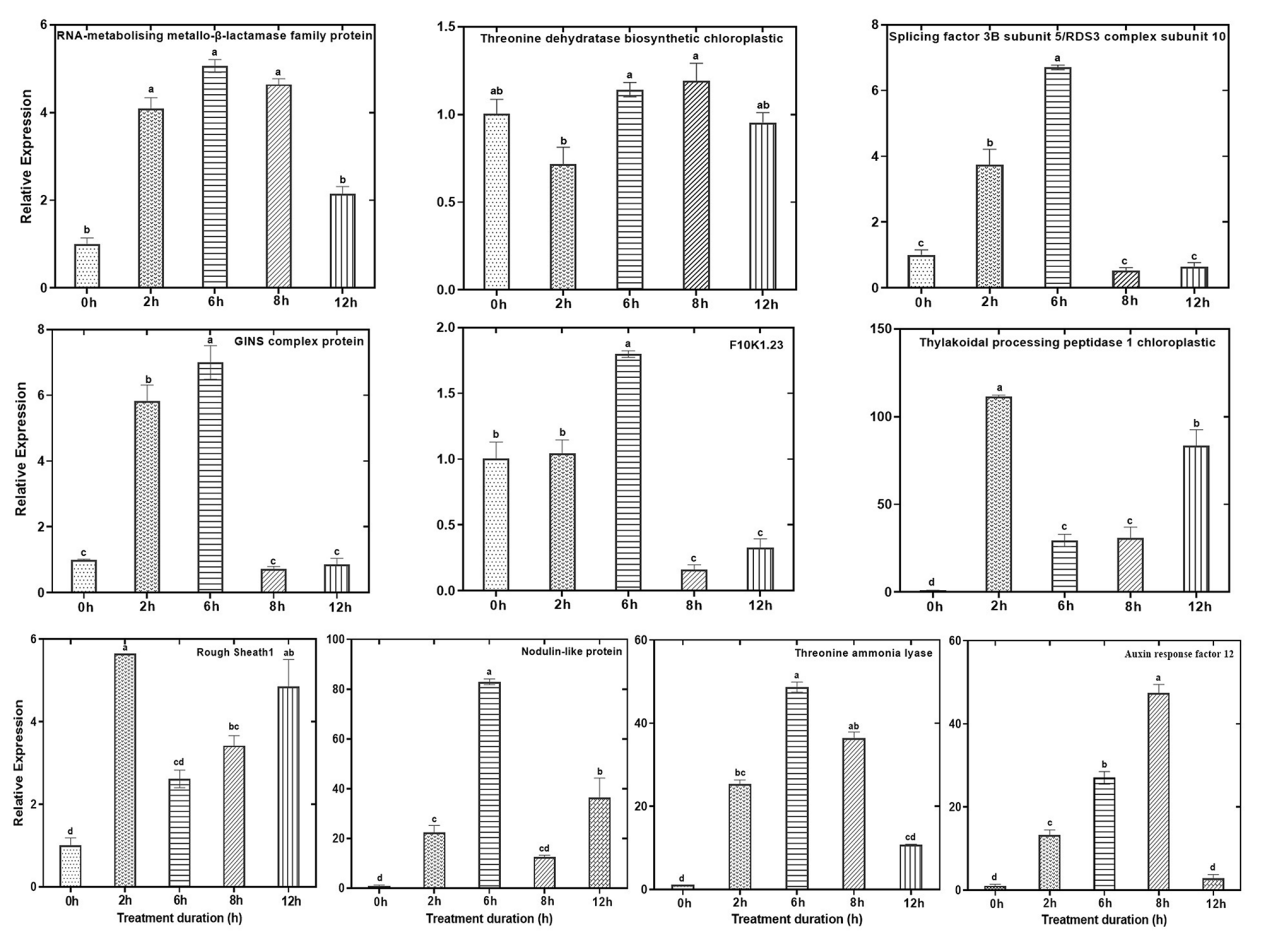
Validation of proteomic data by qRT-PCR in Gurez local genotype. The Y axis represent the ‘relative expression’ and X axis the ‘stress time points’ for all the selected genes from DRPs. Data show the mean ± SD of three replicates, and significant differences between treatment and control samples were indicated by letters at p ≤ 0.05. The internal control used was alpha tubulin and ΔΔCt was calculated using 0 h as control

Expression analysis employing qRT-PCR was used to analyze the transcriptional activities of fifteen randomly selected genes including the three main novel proteins (10 from Gurez local and 5 from GM6) in order to corroborate our proteomic findings. In general, in case of Gurez local, the results showed that 5 up-regulated [RNA-metabolising metallo-beta-lactamase family protein; rough sheath1; threonine dehydratase biosynthetic chloroplastic (reasonably higher expression at 6 and 8 h of LT treatments); thylakoidal processing peptidase 1 chloroplastic; and nodulin-like protein], and 2 down-regulated proteins (GINS complex protein and F10K1.23 showing down-regulation post 6 h LT stress) coincided well with the patterns of the transcript levels of corresponding coding genes at different LT stress time points. However, other 3 proteins (auxin response factor 12, splicing factor 3B subunit 5/RDS3 complex subunit 10, and threonine ammonia-lyase) showed an exponential increment in their corresponding mRNA levels in the first hours of stress, which later on remarkably decreased with the progression of stress treatment (Fig. 10). The fascinating part of the story is that the proteins including threonine dehydratase biosynthetic chloroplastic, thylakoidal processing peptidase 1 chloroplastic, and nodulin-like protein believed to have a novel and potent role in LT stress tolerance in maize as identified in Gurez local were consistent with the proteomic findings, hence strengthening the basis for the set hypothesis.

**Fig. 11:**
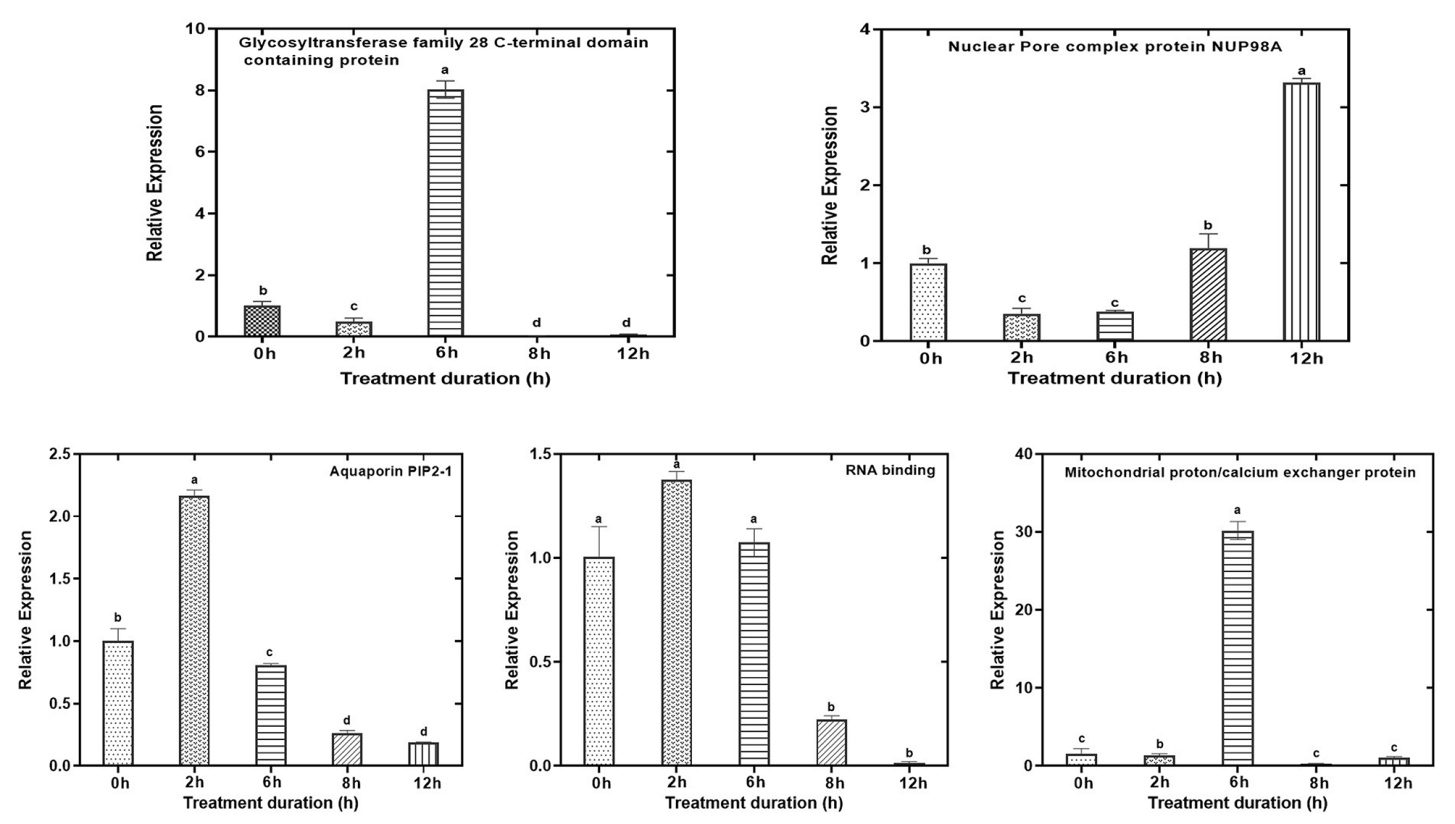
Validation of proteomic data by qRT-PCR for selected genes from DRPs in GM6 maize genotype. Data show the mean ± SD of three replicates, and significant differences between treatment and control samples were indicated by letters at p ≤ 0.05

In case of GM6, among the 5 selected proteins, 3 proteins (mitochondrial proton/calcium exchanger protein, nuclear pore complex protein NUP98A, and RNA binding) were observed to to replicate the qRT-PCR approach while considering the general trend of their transcript expression. Hence, overall expression patterns of the studied genes were found to corroborate with our proteomic findings. However, rest 2 proteins viz. glycosyltransferase family 28 C-terminal domain containing protein, and aquaporin PIP2-1 exhibited opposite trends with their mRNA homologues (Fig. 11).

### Correlation analysis of abundance of selected proteins with their corresponding transcript levels in maize genotypes

**Fig. 12:**
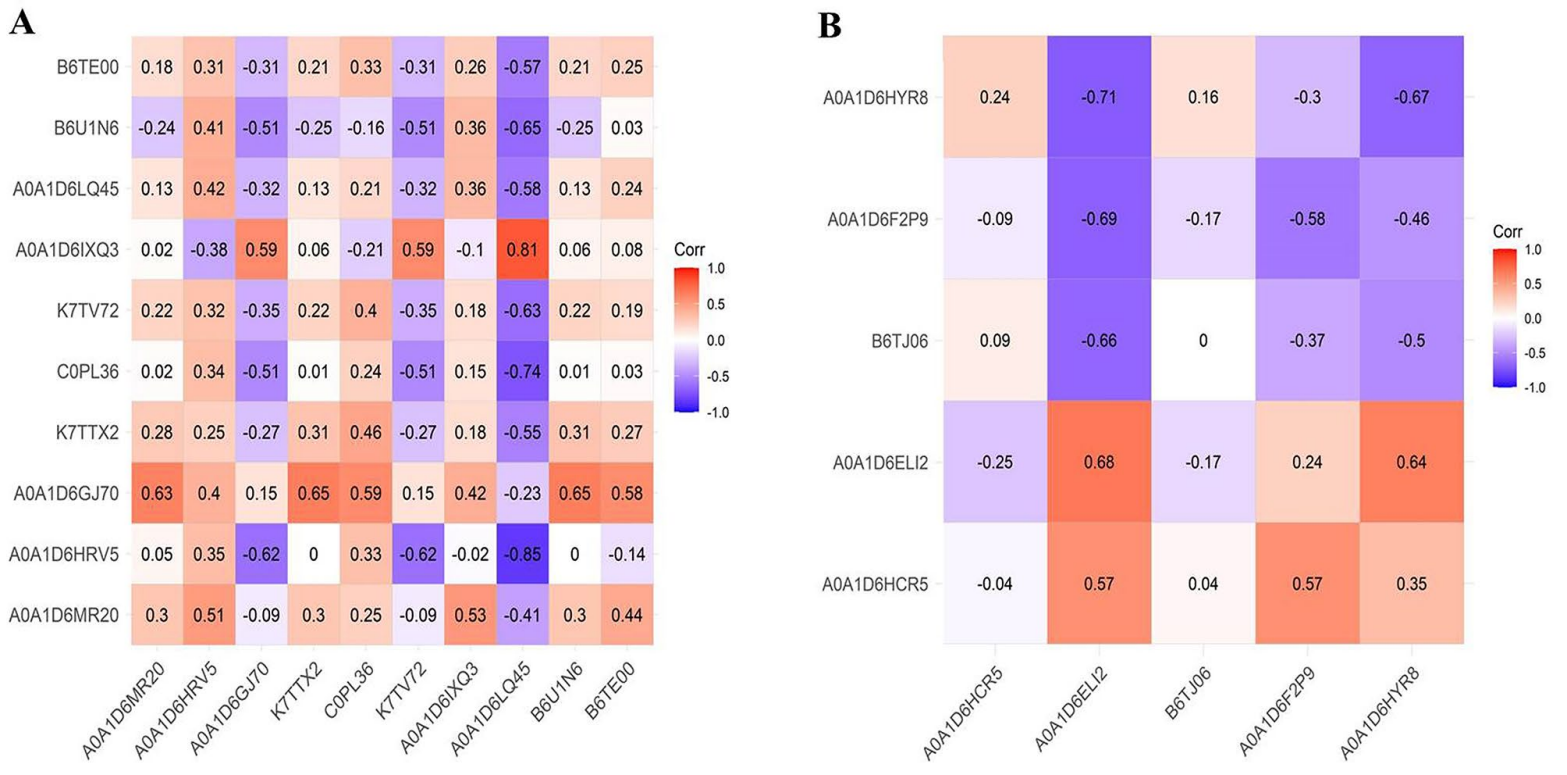
Correlational analysis of selected proteins with their corresponding mRNA levels in **(A)** Gurez local **(B)** GM6. The red color in the scale denotes positive correlation while blue denotes inverse correlation between the two

The correlational study between the levels of selected proteins’ transcripts and their abundance in the two genotypes demonstrated the consistency of gene expression at various stress time points (Fig. 12). Evidently, proteins with uniprot IDs A0A1D6MR20, A0A1D6HRV5, AOA1D6GJ70, K7TTX2, C0PL36, and B6TE00 exhibited a positive correlation with their mRNA levels in the Gurez local genotype. However, the trend was reverse in the rest four proteins bearing IDs K7TV72, A0A1D6IXQ3, A0A1D6LQ45, and B6U1N6. Similarly, in case of GM6, the correlation analysis revealed positive (for protein with uniprot ID of A0A1D6ELI2), slightly negative (uniprot ID: A0A1D6HCR5), considerably negative (uniprot IDs: A0A1D6F2P9 and NUP98A), and no (uniprot ID: B6TJ06) corroboration between the mRNA and protein levels.

## Discussion

LT stress severely affects early vigor and production of maize [38] which in turn signifies the importance of exploring and understanding peculiar molecular networks behind cold and other abiotic stresses. Therefore, unravelling the stress-driven proteome changes in plants holds remarkable significance since proteins, unlike transcripts, are direct effectors of plant responses to stress conditions [39]. In this context, we, therefore, demonstrated the role of various LT induced proteins conferring exceptional LT tolerance to the Gurez local maize genotype from Kashmir Himalaya compared to its susceptible counterpart, GM6. Interestingly, a few proteins showing significant expression levels in Gurez local under LT treatment are reported for the first time for their potent role in conferring LT stress tolerance to maize, however, the hypothesis warrants further investigations. Overall, our comparative proteomic analysis serves as an important exploratory method to determine the impact of environment modulated gene expression under LT stress.

### Differentially regulated proteins in Gurez local

We identified 19 proteins in Gurez local in response to LT stress, among which only two were found to get down-regulated, and rest 17 exhibited remarkable abundance with the progression of LT stress including a few novel proteins. Pertinently, numerous stress responsive proteins with some novel LT-induced ones have been well documented from different species earlier as well [40]. Here, majority of the proteins identified in Gurez local were found to be involved in regulation of seed germination and seedling growth, timing of floral transition, lipid glycosylation, amino acid metabolism, and defense mechanisms in response to LT stress. Based on the involvement of identified proteins in different processes, it may be concluded that Gurez local has developed strong molecular strategies in due course of time, to stand with the harsh environmental cues especially at sprouting stage. Nevertheless, and in light of the limited scope of present investigation, further experimentation is needed to validate the role of identified proteins in different metabolic machineries and through various developmental stages of the plant.

#### Proteins related to seedling establishment in LT tolerant Gurez local

Studies have shown that crop quality and yield is primarily affected by seed germination and seedling establishment [41]. Importantly, our study was also focused on the stress-responsiveness of maize seedlings at three-leaf stage. As a response against LT stress, we found four proteins involved in the process of seedling growth from the LT tolerant genotype of this staple crop. Among these, ‘NHL domain-containing protein (NHL1)’, with established role against pathogen induced stress condition in *Glycine max* at seedling stage [42], was supposed to exhibit possible relation with the seedling establishment in Gurez local as well. Other proteins include ‘GINS complex protein’ and ‘RNA-metabolising metallo-beta-lactamase family protein’. The former has been found to play a role in cell cycle processes in *Arabidopsis* and rice [43] while the members of later in early stages of plant development in *Arabidopsis* [44]. These proteins subsequently result in the overall vegetative growth of the plant through coordinated interaction of cellular cycle and cell expansion machinery. Finally, ‘F10K1.23’, belonging to the family of UDP glycosyltransferases is also believed to promote the seedling growth associated LT tolerance in maize. Very recently, this protein was found to play a critical role in regulating grain size and abiotic stress tolerance with associated metabolic redirection flux in rice [45].

#### Proteins involved in floral transition under LT stress

As soon as the corn seedlings establish themselves towards final vegetative phase (V5) a crucial developmental change in plant’s lifecycle is marked and transition to flowering begins. The flowering time is critical factor in determining the crop yield in maize, and importantly alteration in flowering timing is a strategy used to survive abiotic stresses [46]. In our study, we identified five proteins with a potent role in transition to flowering stage in maize. For instance, the novel protein identified under LT stress viz. ‘Thylakoidal processing peptidase 1 chloroplastic’ is believed to develop the photosynthetic tissues to mark plants’ growth towards flowering stage owing to its documented role in chloroplast development under salt and osmotic stress in wheat seedlings [47]. Similarly, the up-regulation of ‘rough sheath1’ protein known to play an indispensable role in cell division and expansion in maize leaf [48], is also supposed to cause expansion of maize leaves and leaf initiation, heading the plant towards the flowering stage. The ‘translation initiation factor IF-2’ conferring abiotic stress tolerance to *Tamarix hispida* [49] and salt stress tolerance to yeast and plants [50], plays an important role in regulating the protein synthesis necessary for switching the plant from vegetative to reproductive phase. The plant signaling key factor ‘calcium-dependent protein kinase’, otherwise involved in calcium signaling while regulating flowering time in Arabidopsis [51], may also regulate the floral transition in Gurez local under LT stress. Additionally, the ‘splicing factor 3B subunit 5/RDS3 complex subunit 10’ regulating the fall of alfalfa terminal buds [52] might also contribute in the terminal bud growth thereby allowing the transition of Gurez local genotype from juvenile to adult phase. All these proteins, in one way or the other, help the Gurez local to grow and develop under unfavorable LT stress.

#### Transport of sugar moieties and essential amino acids is a vital strategy of LT tolerance in Gurez local

One of the interesting and novel proteins that we identified in Gurez local is ‘nodulin-like protein’ that was found highly abundant under LT treatment in terms of both protein and mRNA content. As the phloem loading/unloading is highly responsive to environmental changes, it is believed to be involved in transport related activities in phloem under LT stress conditions. In fact, previous findings show its potent role in transport of sugar moieties wherein this protein was found to get accumulated in the sieve element plasma membrane of Arabidopsis [53]. Interestingly, in non-nodulating plant species including maize, nodulin like proteins have also been found with their special role in transporter activity throughout the plant developmental stages [54]. Hence, in non-leguminous plants like maize, the functions of ‘nodulin like protein’ are emerging and require further investigations to validate its role in abiotic stress associated phloem transport.

Apart from sugars, majority of the amino acids have a central role in plant environmental stress response [55]. Proteins related to amino acid metabolism, particularly, ‘Asp family amino acids’ that were found in increased abundance in Gurez local were one among the novel proteins viz. ‘Threonine dehydratase biosynthetic chloroplastic’ and ‘Threonine ammonia-lyase’. Besides playing primary role in transport of nitrogen at different stages of development in plants, the ‘Asp family amino acids’ have essential roles in plant abiotic stress response as well [31]. This was reported in a recent study wherein exogenous application of some amino acids on maize under high temperature stress showed positive effect on growth and development of the crop plant [56]. However, the direct involvement of ‘Threonine dehydratase biosynthetic chloroplastic protein’ in stress responsiveness in plants has not been confirmed so far and requires further explorations.

#### Proteins in relation to ‘response to stimuli’ under LT stress induced conditions

Series of defense mechanisms in response to environmental cues operate within an organism necessary for its survival [57], and specific proteins in response to stress per se are produced in huge quantities through a variety of mechanisms [58]. In our study, proteins associated with LT stress tolerance found in higher abundance include the farnesylated protein, auxin response factor 12, the role of which has been highlighted in case of stressed tomato [59] and banana [60]; IND1 (iron-sulfur protein required for NADH dehydrogenase)-like reported earlier in abiotic stress response in rice [61]; Tetratricopeptide repeat (TPR)-like superfamily protein expressed under osmotic stress responses in Arabidopsis [62]; NHL domain-containing protein, the overexpression of which provides resistance to biotic stress in soyabean [42]; Aquaporin PIP2-1 having a full-fledged role in abiotic stress tolerance in plants [63]; and Peroxidase 2-like conferring insect resistance in maize kernels [64]. The present study provides additional evidence that all these proteins contribute to LT tolerance in maize, hence, our findings provide a potent genetic resource for enhancing LT tolerance in this demanding crop plant.

### Differentially regulated proteins in GM6

A differential ability of LT tolerant and LT sensitive maize genotypes to cope with stress conditions is evident from the changes in abundance of different stress protective proteins in Gurez local as compared to its counterpart. The tolerant genotype does not suffer more from energy metabolism disruption and withstands the harsh environmental scenario which has been reported earlier also [65] as compared to the stress-sensitive genotype. The reduced levels of most of the stress protective proteins and alteration in the expression of proteins involved in basic metabolic processes in GM6 reveals the inefficient stress tolerance mechanism in the stress sensitive genotype.

#### Regulation of mRNA/protein nucleo-cytoplasmic trafficking in plant stress response

The nuclear pore complex (NPC) family components not only play key roles in general growth and development of plants but also in response to different stressors affecting the plant survival [66]. The NPC family consisting of different proteins named as nucleoporins (Nups) regulate the molecular trafficking between nucleus and cytoplasm [67]. In the present study, two proteins associated with NPC family were found in abundance under LT stress in GM6. These include nuclear pore complex protein NUP98A, and importin subunit alpha-2, partial. On the other hand, the down-regulation of third protein viz. nuclear pore complex protein NUP155 in LT susceptible genotype depicts the alteration in relative protein abundance due to unfavorable stress conditions. All these findings demonstrate the role of these proteins in NPC-mediated plant stress responses.

Furthermore, the down-regulation of RNA binding protein and mediator of RNA polymerase II transcription subunit 11 by GM6 in response to LT treatment depicts the overall susceptibility of the genotype towards stress conditions, hence revealing the developmental arrest. Similar reports on decreased abundance of most of the stress responsive proteins in stress-sensitive species have been well documented [65] previously. Otherwise, RNA binding proteins associated with post transcriptional regulation of RNA metabolism play central roles in stress responses besides helping the plant growth and development [68].

#### Altered abundance of other critical stress sensitive proteins in GM6

Aquaporins belonging to the class of membrane proteins facilitate water transport and transport of other solutes thereby playing a vital role in cell signaling, nutrient acquisition and stress response [69]. In GM6, aquaporin PIP2-1 was found in higher abundance to ensure the water availability to the plant under stressful conditions, coherent with the previous findings on maize genotypes under drought stress [69]. On the other side, leucine zipper/EFhandcontaining transmembrane protein 1 (LETM1) like protein has a remarkable role in mitochondrial translation in early seed development as studied in *Arabidopsis thaliana* [70]. The higher levels of this protein in GM6 depicts the struggle of this genotype under LT stress required for the re-establishment of mitochondrial homeostasis and repairing of stress induced molecular damage. Interestingly, the reduced levels of another mitochondria associated protein viz. mitochondrial proton/calcium exchanger protein are concomitant with the former one. Actually, one of the death inducing mechanisms in plants under adverse environmental conditions is the mitochondrial permeability transition which is characterized by collapsing of electrochemical gradient across the inner mitochondrial membrane [71]. Hence the probable reason behind the reduction in the protein levels is to prevent the GM6 genotype from mitochondrial damage caused due LT stress, as no special adaptive mechanisms operate in this stress sensitive genotype to withstand LT conditions.

In addition to the above, it is well known fact that ambient temperature profoundly affects the flowering time in plants [72]. So, in case of LT sensitive GM6, the decreased abundance of flowering time control protein FCA-like clearly indicates the delayed flowering transition due to LT stress thereby diminishing the plant growth. Furthermore, enhanced transfer of sugar moieties under abiotic stress conditions has gained much interest during the past decade for their roles in ROS scavenging, signaling and osmoprotectants [73]. In our study also, the elevated levels of glycosyltransferase family 28 C-terminal domain containing protein are believed to have a potential role in transfer of sugar moieties to alleviate the cellular levels of sugars that in turn help the GM6 genotype to survive under adverse environment. Our study provides additional theoretical basis as well as practical significance for further exploration of molecular mechanisms operating in the golden crop, maize, in response to LT stress.

### Positive correlation between proteins and their transcript abundance confirms the reliability of our data

Most of the proteins among those which were selected for qRT-PCR analysis coupled well with their corresponding transcript levels especially the three novel proteins from Gurez local viz. hreonine dehydratase biosynthetic chloroplastic, thylakoidal processing peptidase 1 chloroplastic, and nodulin-like protein identified in our study. The observation was also confirmed by the correlational analysis between the two. However, few proteins whose abundance did not match with transcript levels from both genotypes may be due to differences in the synthesis and decay rates of mRNA and proteins in addition to microRNA regulated protein synthesis [74].

## Conclusion

In the present study, a comprehensive comparative approach was applied to decipher the molecular basis of LT tolerance in Gurez local as compared to GM6. We successfully identified 19 DRPs in Gurez local and only 10 in GM6 in response to LT stress of 12 h duration at early seedling stage. Most of the proteins (17) in Gurez local exhibited up-regulated expression under LT stress while only 2 were found to get down-regulated with the progression of LT treatment. The proteins were associated with the regulation of seed germination and seedling growth, floral transition timing, lipid glycosylation, amino acid metabolism, and defense adaptations in response to LT stress, necessary for the survival under extreme environments during early growth phases. Contrary to this, in LT susceptible GM6, 5 proteins were found in higher abundance and 5 got down-regulated under LT conditions. These identified proteins influence the basic metabolic activities like cell cycle regulation, nucleo-cytoplasmic trafficking and a few in stress defense mechanisms. Most notably, the present study identified the ‘three’ novel proteins from Gurez local, which include threonine dehydratase biosynthetic chloroplastic, thylakoidal processing peptidase 1 chloroplastic, and nodulin-like protein, the roles of which has not been established in plant stress defense so far. The results show that Gurez local may serve as a repository for exploring more LT responsive genes important in molecular breeding of LT tolerant maize genotypes. Furthermore, for most of the proteins identified in Gurez local, the changes in protein abundance were consistent with their corresponding transcript levels, hence, substantiating our proteomic findings. In nutshell, our investigations clarified the strategies employed by temperate grown ‘Gurez local maize’ to withstand adverse low temperatures of Kashmir Himalayas comparative to its tropical grown counterpart and also elucidated the basic molecular networks and metabolic pathways associated with LT tolerance in maize.

## Materials and methods

### Plant material, lt treatment and sampling

Two maize genotypes exhibiting differential temperature tolerance were selected for the experiment. LT tolerant genotype viz., Gurez local (native to Kashmir Himalayas) and LT sensitive, Gujarat-Maize-6 (GM6-native to Gujarat) lines were obtained from IACRP-Maize (SKAUST-K) Srinagar Centre. Seeds were sown in soil: sand mixture (v/v 3:1) in pots (volume 300 ml, diameter 8 cm and height 12 cm) in a completely randomized experimental design and a single seedling was maintained per pot. The seeds were germinated and allowed to grow up to three-leaf stage in a growth chamber (Blue Star, ICo No: NKL-750) maintained at a temperature of 25 ± 2 °C, photoperiod of 16 h light/8 h dark under a relative humidity of 70%. After two weeks, maize seedlings were subjected to LT stress of 6 °C for a period of 12 h wherein 0 h served as the control. Based on our preliminary physio-biochemical investigations on the same samples [9], significant results were seen at some selected hours of stress time points viz., 2 h, 6 h, 8 and 12 h (Fig. 1). Hence, for carrying out the proteomic analysis and quantitative real time PCR (qRT-PCR), plant leaf samples were harvested on these selected hours of stress. So, a total of 5 time-points were selected for carrying out the proteomic analysis in both the genotypes. All methods were carried out in accordance with relevant guidelines.

### Protein sample preparation and 2D-PAGE

Extraction of protein samples was done following the methodology [20] subjected to certain modifications. Briefly, for each time point, 3–5 seedlings (at three-leaf stage) were collected and their leaves (three) were harvested. Latter were pooled and 1 g leaf tissue was ground to fine powder in liquid nitrogen and then dissolved in 10 ml chilled homogenization buffer [sucrose (40%), 50 mM HEPES-KOH (pH, 7.5), β mercaptoethanol (1%), 1 mM EDTA (pH, 7.5), 60 mM sodium fluoride]. After vortexing the homogenates, 15 ml tris-equiliberated phenol was added to each sample. Sample solutions were kept on rocker for 30´ at 4 °C, and then centrifuged (5000 x g at 4 °C) for 15 min. Supernatants (phenol phase) were transferred to clean and pre-chilled tubes, and 0.1 M ammonium acetate in methanol was added for protein precipitation at -20 °C overnight. The samples were centrifuged (10,000 x g at 4 °C) for 25 min and pellets were washed three times with 80% acetone and then resuspended in 500 µl rehydration buffer on ice.

Prior to isoelectric focusing (IEF), protein samples (500 µg) were subjected for active rehydration overnight at 25 °C into a 24 cm GE Healthcare Strip Holder. First, the protein samples were diluted with 2-D rehydration buffer [8 M urea, 2 M thiourea, 2% CHAPS (w/v), 20 mM DTT, 0.5% pharmalyte (v/v, pH 4 − 7) and 0.05% bromophenol blue (w/v)], and 450 µL of the diluted proteins were used to rehydrate the strips passively. The rehydrated strips (24 cm, pH 4–7) were subjected to IEF using Ettan IPGphor system (GE Healthcare, USA) under the following standardized 18 h program: 200 v for 1 h, 500 v for 1 h, 1000 v for 2 h each for gradient and step, and 10,000 v for 2 h gradient with 8 h step. The focused strips were equilibrated in 15 mL of equilibration buffer [6 M urea, 50 mM Tris-HCl (pH 8.8), glycerol (30%, v/v), and SDS (2%, w/v)] first by reduction with DTT, followed by alkylation with iodoacetamide (2.5%, w/v) in the same buffer, each for 10 min.

The second dimension was conducted by loading the IPG strips onto the 12% SDS polyacrylamide gels. On the top of 1 mm thick 2D gel, the equilibrated strips were sealed using 0.5% low-melting agarose in SDS- electrophoresis buffer (25 mM Tris, free base, 200 mM glycine, and 0.1% SDS). The proteins were allowed to get resolved till the bromophenol blue front reached the gel end on Ettan Dalt-6 electrophoresis unit (GE Healthcare, USA) at a constant voltage of 90 V.

### Gel image analysis and proteome profile evaluation

Gel staining was done using Coomassie Brilliant Blue (CBB) G-250 (Bio-Rad, USA) in a gel staining solution (10% GAA, 45% methanol with 0.3% CBB). Proteins were visualized after 24 h by destaining the 2D gels. Digital images of all the gels were obtained using ImageMaster 2D Platinum (GE Healthcare, USA). Three replicate gel images were combined to create a ‘master gel’ for each stress time-point. The matchset was generated by comparing the master gels from each time-point obtained through pairwise comparisons.

After background subtraction, and spot detection, matched spots were normalized with the help of total density index of the gel images. The proteins showing a change > 2 folds were treated as differentially accumulated.

### Spot excision and In-Gel trypsin digestion

After proper washing of gel slabs, the selected spots were excised using clean scalpel. The gel pieces were cut into roughly 1 mm^3^ cubes and put in clean 1.5 ml eppendorf tubes. After that, de-staining of gel slices was carried out using water followed by 100 µl of 100 mM ammonium bicarbonate. Later on, reduction and alkylation of proteins was completed with 10 mM DTT and 55 mM iodoacetamide, followed by drying of samples in vacuum centrifuge.

Trypsin digestion of all the samples was executed by rehydrating the gel pieces with 30 µl of digestion buffer containing 50 mM NH_4_HCO_3_ and 20 ng/µl of trypsin for 30 min at 4 °C. After required quantity of digestion buffer was absorbed by the proteins, the excess gel enzyme solution was removed. Then, 100 µl 50 mM NH_4_HCO_3_ buffer was added to cover the gel and incubated overnight at 37 °C. After overnight incubation, the digest was recovered to a new tube and formic acid was added to the each tube to stop enzymatic reaction. The final extraction of peptides from gels was carried out and sample peptides were resolubilized in 10–20 µl of 0.1% of formic acid to proceed for MALDI-TOF/TOF analysis, until then dried samples were stored at -20 °C.

### Protein species identification by MALDI-TOF MS

All protein digest samples (protein digests) in lyophilized condition were dissolved with 10 µl dissolving solvents (70% H_2_O, 30% acetone and 0.1% TFA). A suitable matrix solution of ά-cyano-4-hydroxy- cinnamic acid was prepared (6.0 mg of CHCA, 50% water, 50% acetonitrile, and 0.1% TFA). Both matrix and sample solutions were mixed in a 1:1 ratio and were spotted in MALDI plate (Opti-TOF™ 384 Well Insert (123 × 81 mm), MDS Sciex) in dried droplet method. Co-crystallized matrix and sample were allowed to air dry and then subjected for MS/MS analysis by 4800 Plus MALDI-TOF/TOF Analyzer (AB Sciex Pte Ltd.). MALDI-MS and MS/MS spectrum were obtained using standard operating software 4000 Series Explorer™ Remote Client. Instrument was calibrated (plate model) for both MS and MS/MS in reflectron mode against the standard procedures using calibration mixture of 6 known peptides [des-Arg1-Bradykinin, Angiotensin 1, Glu 1-Fibrinopeptide, ACTH(1–17), ACTH(18–39), ACTH III(7–38 )]. MALDI-Spectrum was acquired using laser Nd-YAG which operates at 355 nm to ionize samples. A fixed laser intensity of 6000 and 7000 Hz was used for MS and MS/MS analysis, respectively to ionize the samples. All raw mass spectrometry data in the form of LC-MS/MS files were transformed into mgf (mascot generated files) using Mascot distiller software (www.matrixscience.com). Protein identification was done through maize protein database (UP7305_Z_ mays (AA); accessed on 02 December 2021) using the Mascot search engine (version 2.3.02; Matrix Science, London, UK). Following search parameters were set while performing the identification: taxonomy as ‘other green plants’, cleavage enzyme used trypsin, 1 maximum missed cleavages allowed, carbamidomethyl (C) as fixed modifications and oxidation (M) as variable modifications. The results were filtered at a significance threshold of P < 0.05 combining evidence from individual peptides generated through Mascot. The identified peptide sequences were queried against BLASTp-NCBI (protein-protein BLAST with E-value cut-off of 1e-10) under RefSeq protein of *Zea mays* (https://blast.ncbi.nlm.nih.gov/Blast.cgi?PAGE=Proteins; *Zea mays* taxid: 4577) for further identification and annotation of identified maize proteins.

### Bioinformatic analysis of differentially expressed proteins

Beginning with protein structure prediction, 3D models for each identified protein was developed using Phyre2 web portal for protein modeling, prediction and analysis.

[21]. This algorithm applies hidden Markov models (HMMs) for classification of proteins based on their amino acid sequence, hence predicting the presence of a specific protein domain. For analysis of stereochemical quality of each protein, PDBsum web server was used [22]. Furthermore, enrichment analysis of all proteins was carried out through ShinyGO graphical tool. ShinyGO provides in-depth analysis of gene lists and their characteristics, with graphical visualization of enrichment, pathway and protein interactions [23]. For understanding the system-level of cellular processes under LT stress, protein-protein interaction was studied using STRING database (Search Tool for Retrieval of Interacting Genes/Proteins; Version 11.0; [24]. In addition, online web server CLAP, a useful protein-clustering tool, was employed for classification of identified protein sequences [25]. Blast2GO (now OmicsBox), a research tool for performing high quality functional annotation [26] and for classification of proteins on the basis of molecular functions, biological processes and cellular components was exercised to generate gene ontology representation. Heatmap for visualization of hierarchal clustering of proteomics data and correlation analysis of transcript levels and corresponding protein abundance was generated using R (www.r-project.org/).

### RNA extraction and qRT-PCR

Total RNA extraction from all the selected LT stress time points was performed using Trizol reagent (Invitrogen, Carlsbad, USA) and then reverse transcribed using Revert Aid First Strand cDNA Synthesis kit (Thermo-Fisher Scientific) as per the manufacturers’ recommendations. Primers for selected candidate proteins were designed using Primer 3 (http://bioinfo.Ut.ee/primer3-0.4.0/) software and were purchased from Integrated DNA Technologies (IDT), USA (Table S1). The subsequent qRT-PCR analysis was done as previously described [9] while using 0 h as control against the different treatment time points including 2 h, 6 h, 8 h, and 12 h.

### Statistical analysis

The experimental design followed in the study was completely randomized Design (CRD) with three replications. The qRT-PCR data are presented as mean ± SD of the three biological samples with three technical replicates for each and analysed by GraphPad Prism for Windows Version 5.0 (Graph pad Software San Diego, CA, USA) and Statistix 10 (Analytical software, 2013) following one-way ANOVA. Post-hoc test employed was Tukey’s multiple comparison tests among the means. Results were considered to be significant at p values less than 0.05 (p < 0.05) and the significant differences between control and treated samples were indicated by letters.

## Electronic supplementary material

Below is the link to the electronic supplementary material.

Supplementary Material 1

## Data Availability

The mass spectrometry proteomics data have been deposited to the ProteomeXchange Consortium via the PRIDE (http://www.ebi.ac.uk/pride) partner repository with the dataset identifier PXD036246 (project accession) and 10.6019/PXD036246 (project DOI). Note: The data is currently private, and can be accessed with reviewer account that has been created (Username: reviewer_pxd036246@ebi.ac.uk; Password: OYD2kgKf). It is only after paper is accepted, the PRIDE is to be notified and data will be public.

## References

[1] Raza A, Razzaq A, Mehmood SS, Zou X, Zhang X, Lv Y, Xu J (2019). Impact of climate change on crops adaptation and strategies to tackle its outcome: a review. Plants.

[2] Zeng R, Li Z, Shi Y, Fu D, Yin P, Cheng J (2021). Natural variation in a type-A response regulator confers maize chilling tolerance. Nat Commun.

[3] Waqas MA, Wang X, Zafar SA, Noor MA, Hussain HA, Azher Nawaz M, Farooq M (2021). Thermal stresses in maize: effects and management strategies. Plants.

[4] Long SP, Ort DR (2010). More than taking the heat: crops and global change. Curr Opin Plant Bio.

[5] Leipner J, Stamp P. Chilling stress in maize seedlings. Handbook of maize: its biology. 2009; (pp. 291–310).Springer, New York, NY.

[6] Sobkowiak A, Jończyk M, Adamczyk J, Szczepanik J, Solecka D, Kuciara I, Sowiński P (2016). Molecular foundations of chilling-tolerance of modern maize. BMC Genomics.

[7] Ramazan S, Bhat HA, Zargar MA, Ahmad P, John R (2021). Combined gas exchange characteristics, chlorophyll fluorescence and response curves as selection traits for temperature tolerance in maize genotypes. Photo Res.

[8] Lamari N, Zhendre V, Urrutia M, Bernillon S, Maucourt M, Deborde C, Moing A (2018). Metabotyping of 30 maize hybrids under early-sowing conditions reveals potential marker-metabolites for breeding. Metabolomics.

[9] Salika, R., Riffat, J. Abiotic stress responses in maize: a review. Acta Physiol Plant 2021;43:130. 10.1007/s11738-021-03296-0

[10] Sowiński P, Fronk J, Jończyk M, Grzybowski M, Kowalec P, Sobkowiak A. Maize response to low temperatures at the gene expression level: a critical survey of transcriptomic studies.Front Plant Sci. 2020;1488. 10.3389/fpls.2020.576941 PMC7550719 33133117

[11] Yu T, Zhang J, Cao J, Cai Q, Li X, Sun Y, Duan Y (2021). Leaf transcriptomic response mediated by cold stress in two maize inbred lines with contrasting tolerance levels. Genomics.

[12] Li H, Yang M, Zhao C, Wang Y, Zhang R (2021). Physiological and proteomic analyses revealed the response mechanisms of two different drought-resistant maize varieties. BMC Plant Biol.

[13] Zhou G, Wang J, Zhang X, Guo M, Yu G (2020). Predicting functions of maize proteins using graph convolutional network. BMC Bioinform.

[14] Cheng L, Wang Y, He Q, Li H, Zhang X, Zhang F (2016). Comparative proteomics illustrates the complexity of drought resistance mechanisms in two wheat (*Triticum aestivum* L.) cultivars under dehydration and rehydration. BMC Plant Biol.

[15] Xu E, Chen M, He H, Zhan C, Cheng Y, Zhang H, Wang Z (2017). Proteomic analysis reveals proteins involved in seed imbibition under salt stress in rice. Front Plant Sci.

[16] Méchin V, Balliau T, Château-Joubert S, Davanture M, Langella O, Négroni L, Damerval C (2004). A two-dimensional proteome map of maize endosperm. Phytochem.

[17] Zhang XL, SI BW, Fan CM, LI HJ, Wang XM (2014). Proteomics identification of differentially expressed leaf proteins in response to*Setosphaeria turcica*infection in resistant maize. J Integr Agric.

[18] Dworak A, Nykiel M, Walczak B, Miazek A, Szworst-Łupina D, Zagdańska B, Kiełkiewicz M (2016). Maize proteomic responses to separate or overlapping soil drought and two-spotted spider mite stresses. Planta.

[19] Vidal N, Barbosa H, Jacob S, Arruda M (2015). Comparative study of transgenic and non-transgenic maize (Zea mays) flours commercialized in Brazil, focusing on proteomic analyses. Food chem.

[20] Subba P, Barua P, Kumar R, Datta A, Soni KK, Chakraborty S, Chakraborty N (2013). Phosphoproteomic dynamics of chickpea (*Cicer arietinum* L.) reveals shared and distinct components of dehydration response. J Proteome Res.

[21] Kelley LA, Mezulis S, Yates CM, Wass MN, Sternberg MJ (2015). The Phyre2 web portal for protein modeling, prediction and analysis. Nat.

[22] Laskowski RA, Jabłońska J, Pravda L, Vařeková RS, Thornton JM, PDBsum (2018). Structural summaries of PDB entries. Protein Sci.

[23] Ge SX, Jung D, Yao R (2020). ShinyGO: a graphical gene-set enrichment tool for animals and plants. Bioinform.

[24] Szklarczyk D, Gable AL, Lyon D, Junge A, Wyder S, Huerta-Cepas J, Mering CV (2019). STRING v11: protein–protein association networks with increased coverage, supporting functional discovery in genome-wide experimental datasets. Nucleic acids Res.

[25] Gnanavel M, Mehrotra P, Rakshambikai R, Martin J, Srinivasan N, Bhaskara RM (2014). CLAP: a web-server for automatic classification of proteins with special reference to multi-domain proteins. BMC Bioinform.

[26] Conesa A, Götz S, García-Gómez JM, Terol J, Talón M, Robles M (2005). Blast2GO: a universal tool for annotation, visualization and analysis in functional genomics research. Bioinform.

[27] Bäurle I, Dean C (2006). The timing of developmental transitions in plants. Cell.

[28] Fan T, Li X, Yang W, Xia K, Ouyang J, Zhang M (2015). Rice osa-miR171c mediates phase change from vegetative to reproductive development and shoot apical meristem maintenance by repressing four OsHAM transcription factors. PLoS ONE.

[29] Strasser R, Seifert G, Doblin MS, Johnson KL, Ruprecht C, Pfrengle F, Estevez JM. Cracking the “Sugar Code”: A snapshot of N-and O-glycosylation pathways and functions in plants cells.Front Plant Sci. 2021; 12. 10.3389/fpls.2021.640919 PMC7933510 33679857

[30] Aslam M, Fakher B, Ashraf MA, Cheng Y, Wang B, Qin Y (2022). Plant low-temperature stress: signaling and response. Agronomy.

[31] Han M, Zhang C, Suglo P, Sun S, Wang M, Su T (2021). L-Aspartate: an essential metabolite for plant growth and stress acclimation. Molecules.

[32] Bhusal B, Poudel MR, Rishav P, Regmi R, Neupane P, Bhattarai K, Acharya S (2021). A review on abiotic stress resistance in maize (*Zea mays* L.): effects, resistance mechanisms and management. J Biol Todays World.

[33] Hao W, Collier SM, Moffett P, Chai J (2013). Structural basis for the interaction between the potato virus X resistance protein (rx) and its cofactor ran GTPase-activating protein 2 (RanGAP2). J Biol Chem.

[34] Owttrim GW (2006). RNA helicases and abiotic stress. Nucleic acids Res.

[35] Kazan K, Lyons R (2006). The link between flowering time and stress tolerance. J Exp Bot.

[36] Vishwakarma K, Mishra M, Patil G, Mulkey S, Ramawat N, Pratap Singh V, Sharma S (2019). Avenues of the membrane transport system in adaptation of plants to abiotic stresses. Crit Rev Biotechnol.

[37] Dourmap C, Roque S, Morin A, Caubrière D, Kerdiles M, Béguin K, Couée I (2020). Stress signalling dynamics of the mitochondrial electron transport chain and oxidative phosphorylation system in higher plants. Ann Bot.

[38] Zeng R, Li Z, Shi Y, Fu D, Yin P, Cheng J, Yang (2021). Natural variation in a type-A response regulator confers maize chilling tolerance. Nat Commun.

[39] Dong A, Yang Y, Liu S, Zenda T, Liu X, Wang Y, Duan H (2020). Comparative proteomics analysis of two maize hybrids revealed drought-stress tolerance mechanisms. Biotechnol Biotechnol Equip.

[40] Gao F, Zhou Y, Zhu W, Li X, Fan L, Zhang G (2009). Proteomic analysis of cold stress-responsive proteins in Thellungiella rosette leaves. Planta.

[41] Tian Y, Guan B, Zhou D, Yu J, Li G, Lou Y. Responses of seed germination, seedling growth, and seed yield traits to seed pretreatment in maize (*Zea mays* L.).Sci World J. 2014. 10.1155/2014/834630 PMC4100373 25093210

[42] Maldonado A, Youssef R, McDonald M, Brewer E, Beard H, Matthews B (2014). Overexpression of four Arabidopsis thaliana NHL genes in soybean (Glycine max) roots and their effect on resistance to the soybean cyst nematode (*Heterodera glycines*). Physiol Mol Plant Pathol.

[43] Shultz RW, Tatineni VM, Hanley-Bowdoin L, Thompson WF. Genome-wide analysis of the core DNA replication machinery in the higher plants Arabidopsis and rice. Plant Physiol. 2007 Aug;144(4):1697–714. 10.1104/pp.107.101105 PMC1949880 17556508

[44] Xu R, Ye X, Li QQ. AtCPSF73-II gene encoding an Arabidopsis homolog of CPSF 73 kDa subunit is critical for early embryo development.Gene. 2004 Jan7;324:35–45. 10.1016/j.gene.2003.09.025 14693369

[45] Dong NQ, Sun Y, Guo T, Shi CL, Zhang YM, Kan Y, Lin HX (2020). UDP-glucosyltransferase regulates grain size and abiotic stress tolerance associated with metabolic flux redirection in rice. Nat Commun.

[46] Song K, Kim HC, Shin S, Kim KH, Moon JC, Kim JY, Lee BM (2017). Transcriptome analysis of flowering time genes under drought stress in maize leaves. Front Plant Sci.

[47] Zhu D, Luo F, Zou R, Liu J, Yan Y (2021). Integrated physiological and chloroplast proteome analysis of wheat seedling leaves under salt and osmotic stresses. J Proteom.

[48] Schneeberger RG, Becraft PW, Hake S, Freeling M (1995). Ectopic expression of the knox homeo box gene rough sheath1 alters cell fate in the maize leaf. Genes Dev.

[49] Yang G, Yu L, Wang Y, Wang C, Gao C (2017). The translation initiation factor 1A (TheIF1A) from *Tamarix hispida* is regulated by a Dof transcription factor and increased abiotic stress tolerance. Front Plant Sci.

[50] Rausell A, Kanhonou R, Yenush L, Serrano R, Ros R (2003). The translation initiation factor eIF1A is an important determinant in the tolerance to NaCl stress in yeast and plants. Plant J.

[51] Shi S, Li S, Asim M, Mao J, Xu D, Ullah Z, Liu G, Wang Q, Liu H. The Arabidopsis calcium-dependent protein kinases (CDPKs) and their roles in plant growth regulation and abiotic stress responses.International journal of molecular sciences. 2018 Jun28;19 (7):1900. 10.3390/ijms19071900 PMC6073581 29958430

[52] Du H, Shi Y, Li D, Fan W, Wang Y, Wang G, Wang C (2018). Proteomics reveals key proteins participating in growth difference between fall dormant and non-dormant alfalfa in terminal buds. J Proteom.

[53] Khan JA, Wang Q, Sjolund RD, Schulz A, Thompson GA (2007). An early nodulin-like protein accumulates in the sieve element plasma membrane of Arabidopsis. Plant Physiol.

[54] Denancé N, Szurek B, Noël LD. (2014). Emerging functions of nodulin-like proteins in non-nodulating plant species. Plant Cell Physiol. 2014; 55(3): 469–474. 10.1093/pcp/pct198 24470637

[55] Trovato M, Funck D, Forlani G, Okumoto S, Amir R. Amino Acids in Plants: Regulation and Functions in Development and Stress Defense.Front Plant Sci. 2021; 12. 10.3389/fpls.2021.772810 PMC8559698 34733310

[56] Matysiak K, Kierzek R, Siatkowski I, Kowalska J, Krawczyk R, Miziniak W (2020). Effect of exogenous application of amino acids L-arginine and glycine on maize under temperature stress. Agronomy.

[57] Nejat N, Mantri N. Plant immune system: crosstalk between responses to biotic and abiotic stresses the missing link in understanding plant defence. Curr Issues Mol Biol. 2017 Jul;23(1):1–6. 10.21775/cimb.023.001 28154243

[58] Zhang H, Zhu J, Gong Z, Zhu JK. Abiotic stress responses in plants. Nat Rev Genet. 2022 Feb;23(2):104–19. 10.1038/s41576-021-00413-0 34561623

[59] Bouzroud S, Gouiaa S, Hu N, Bernadac A, Mila I, Bendaou N, Zouine M (2018). Auxin response factors (ARFs) are potential mediators of auxin action in tomato response to biotic and abiotic stress (*Solanum lycopersicum*). PLoS ONE.

[60] Hu W, Zuo J, Hou X, Yan Y, Wei Y, Liu J, Jin Z (2015). The auxin response factor gene family in banana: genome-wide identification and expression analyses during development, ripening, and abiotic stress. Front Plant Sci.

[61] Liang X, Qin L, Liu P, Wang M, Ye H (2014). Genes for iron–sulphur cluster assembly are targets of abiotic stress in rice, *Oryza sativa*. Plant Cell Environ.

[62] Rosado A, Schapire AL, Bressan RA, Harfouche AL, Hasegawa PM, Valpuesta V, Botella MA (2006). The Arabidopsis tetratricopeptide repeat-containing protein TTL1 is required for osmotic stress responses and abscisic acid sensitivity. Plant Physiol.

[63] Afzal Z, Howton TC, Sun Y, Mukhtar MS (2016). The roles of aquaporins in plant stress responses. J Develop Biol.

[64] López-Castillo LM, González-Leyzaola A, Diaz-Flores-Rivera MF, Winkler R, Wielsch N, García-Lara S. Modulation of aleurone peroxidases in kernels of insect-resistant maize (*Zea mays* L.; Pob84-C3R) after mechanical and insect damage.Front Plant Sci. 2020;781. 10.3389/fpls.2020.00781 PMC7300834 32595673

[65] Kosová K, Vítámvás P, Prášil IT (2014). Proteomics of stress responses in wheat and barley—search for potential protein markers of stress tolerance. Front Plant Sci.

[66] Yang Y, Wang W, Chu Z, Zhu JK, Zhang H. (2017). Roles of nuclear pores and nucleo-cytoplasmic trafficking in plant stress responses. Front Plant Sci. 2017; 8: 574. 10.3389/fpls.2017.00574 PMC5388774 28446921

[67] Xu XM, Meier I (2008). The nuclear pore comes to the fore. Trends Plant Sci.

[68] Lee K, Kang H (2016). Emerging roles of RNA-binding proteins in plant growth, development, and stress responses. Mol cells.

[69] Ovrutska II, Kordyum EL. PIP2; 1 aquaporin gene expression in maize hybrids different for drought tolerance to water deficit. Доповіді НАН Украïни; 2019.

[70] Zhang B. Functional characterization of mitochondrial stress response in *Arabidopsis thaliana* (Doctoral dissertation, University of Western Australia). 2012.

[71] Gutiérrez-Aguilar M (2020). Mitochondrial calcium transport and permeability transition as rational targets for plant protection. Biochim et Biophys Acta (BBA)-Bioenergetics.

[72] Jung JH, Seo PJ, Ahn JH, Park CM (2012). Arabidopsis RNA-binding protein FCA regulates microRNA172 processing in thermosensory flowering. J Biol Chem.

[73] Gangola MP, Ramadoss BR. Sugars play a critical role in abiotic stress tolerance in plants. Biochemical, physiological and molecular avenues for combating abiotic stress tolerance in plants. 2018; (pp. 17–38).Academic Press.

[74] Payne SH (2015). The utility of protein and mRNA correlation. Trends Biochem Sci.

