# Supplementary material for: Comparative protein analysis of two maize genotypes with contrasting tolerance to low temperature
Source: BMC Plant Biol. 2023 Apr 5;23:183. doi: 10.1186/s12870-023-04198-8 (PMC10074880; doi:10.1186/s12870-023-04198-8)
Supplement: Supplementary file 1 — Supplementary Material 1 [file 12870_2023_4198_MOESM1_ESM.docx]

**Comparative protein analysis of two maize genotypes with contrasting tolerance to low temperature**

Salika Ramazan, Nelofer Jan and Riffat John*

Plant Molecular Biology Lab, Department of Botany, University of Kashmir, Srinagar -190 006, Kashmir, India

*Corresponding author

Dr. Riffat John

Sr. Assistant Professor

Department of Botany, University of Kashmir

Srinagar – 190 006, Kashmir, India

E-mail: riffatminhaj@kashmiruniversity.ac.in


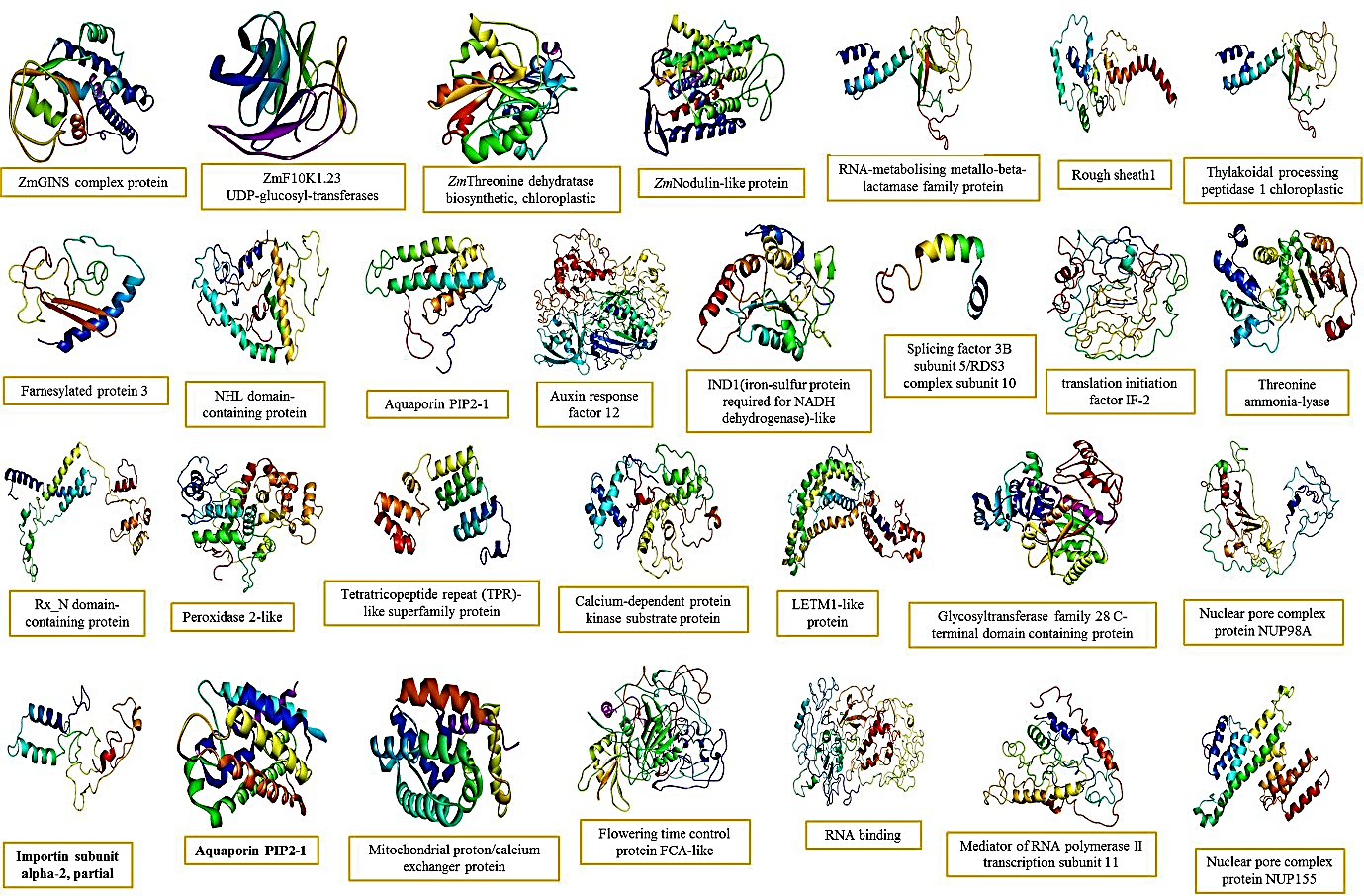
**Fig. S1** Proteins models of all the identified proteins in Gurez local and GM6.


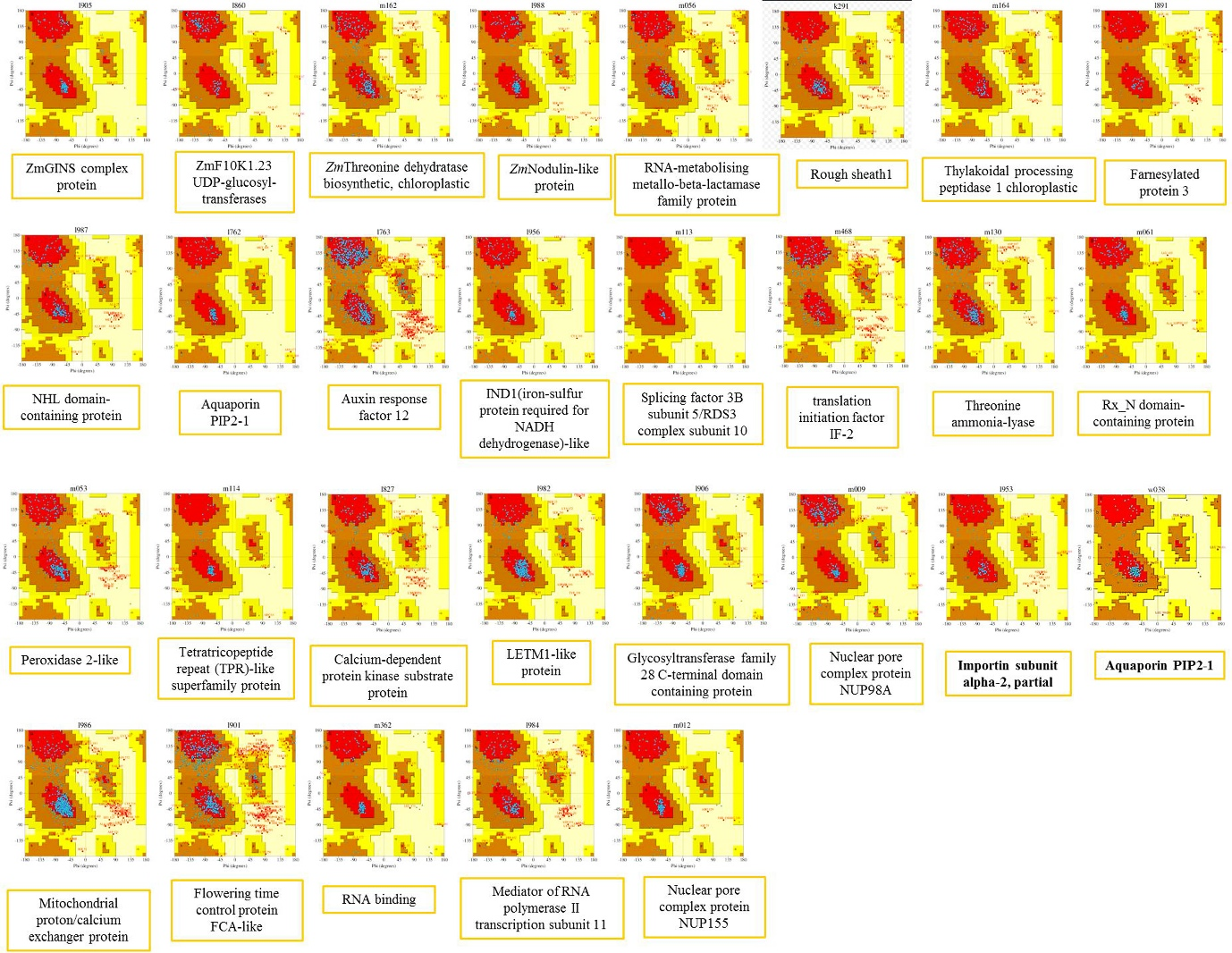


**Fig. S2** Ramachandran plots for validation of proteins models of all identified proteins in Gurez local and GM6.

**
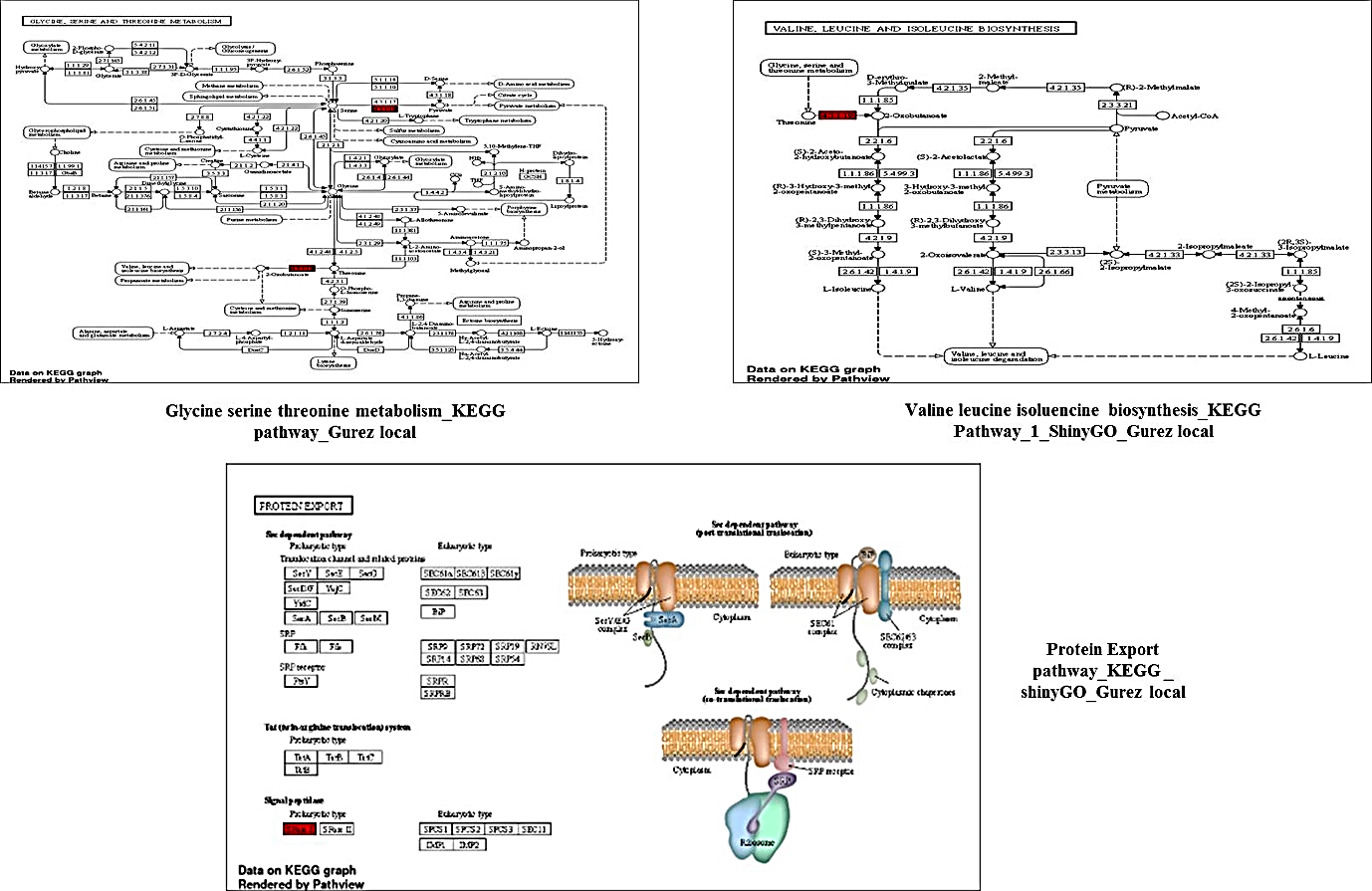
**

**Fig. S3** Changed metabolic pathways in LT treated Gurez local seedlings. Red boxes indicate the increased proteins under LT conditions.

**
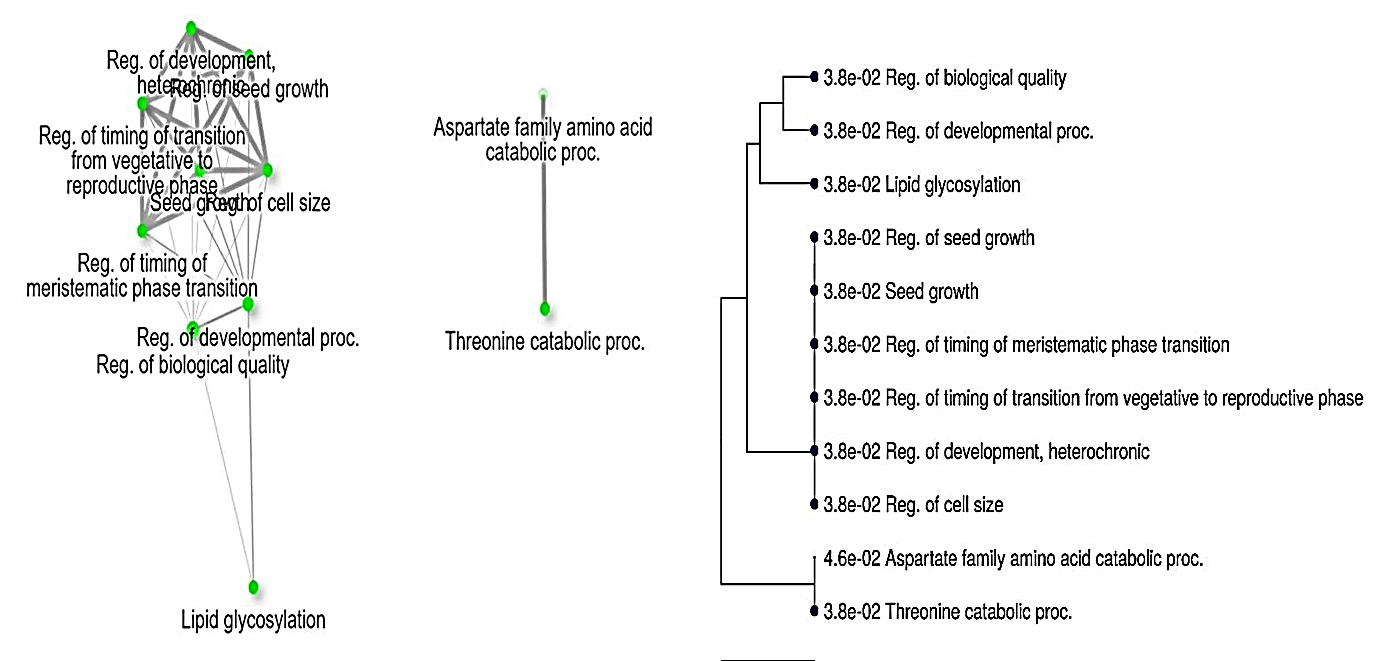
**

**Fig. S4** Interaction of network of enriched pathways and their hieracrchiel clustering in LT tolerant, Gurez local.

**
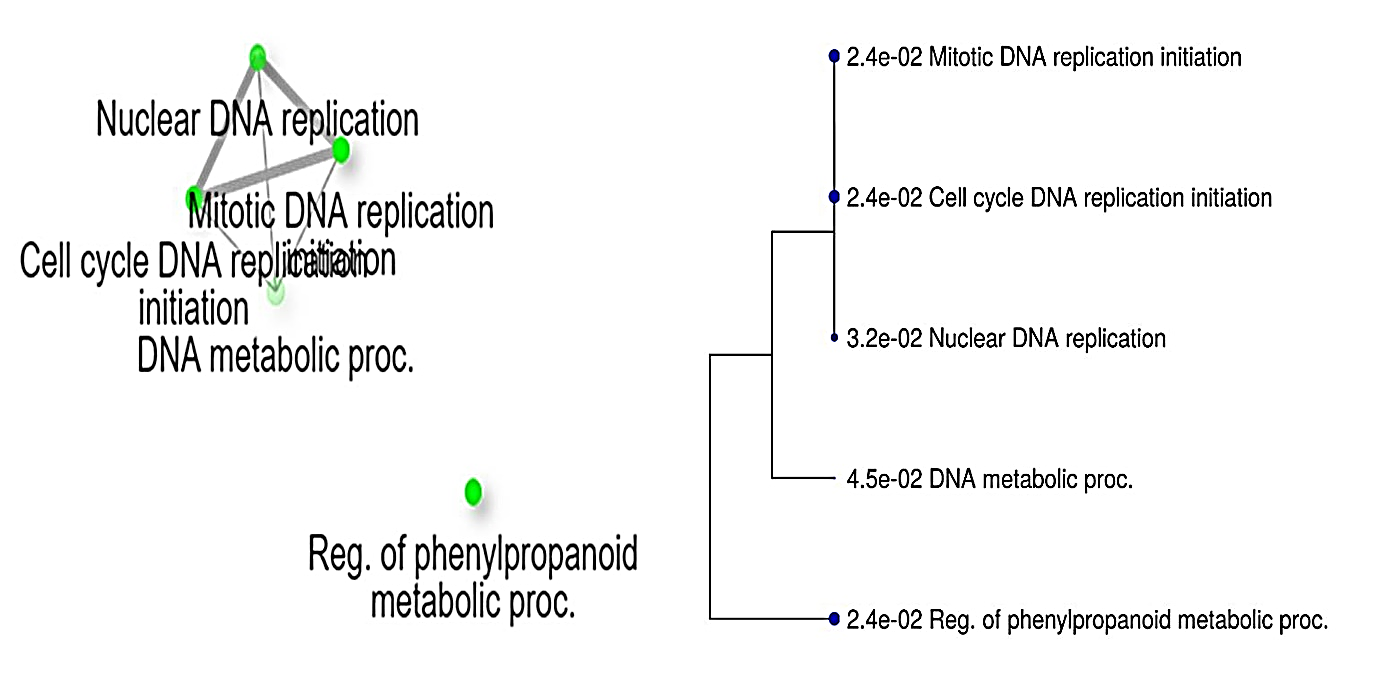
**

**Fig. S5** Interaction of network of enriched pathways and their hieracrchiel clustering in LT sensitive, GM6.

**Table S1:** qRT-PCR primer sequences with NCBI gene accession numbers used in the study

| **Gene** | **NCBI Accession Number** | **Tm** | **Forward (5^/^-3^/^)** | **Reverse (5^/^-3^/^)** |
| --- | --- | --- | --- | --- |
| RNA-metabolising metallo-beta-lactamase family protein  rough sheath1  Threonine dehydratase biosynthetic chloroplastic  Thylakoidal processing peptidase 1 chloroplastic  Auxin response factor 12  Splicing factor 3B subunit 5/RDS3 complex subunit 10  GINS complex protein  F10K1.23  Threonine ammonia-lyase  Nodulin-like protein  Mitochondrial proton/calcium exchanger protein  RNA binding  Glycosyltransferase family 28 C-terminal domain containing protein  Aquaporin PIP2-1  Nuclear pore complex protein NUP155  Alpha-tubulin | ONM31447.1  NM_001111861.2  XM_008646352.3  NM_001147718.1  NP_001349277.1  ONM04429.1  XM_008677942.2  NM_001143247.1  CF349146.1 EST0712  NM_001375357.1  XM_008647687.4  NM_001175827.1  NM_001156409.1  AY243801.1  NM_001324127.1  X73980.1 | 62^o^C  62^o^C  61^o^C  62^o^C  62^o^C  62^o^C  62^o^C  62^o^C  62^o^C  62^o^C  61^o^C  62^o^C  61^o^C  62^o^C  63^o^C  60^o^C | TACCTGTGGGAGAGCTTCA  CCAGAGGAAACGGCACTGGA  TGGATGCATTCAGCCCTCGT  GGAGCATTACGCAGCAGCAAC  ACAACGACCAGAAGCAGAAG  AGCGACAGGTTCAACATCAA  ACCTCGATCGCACGCTCTTC  GGCTACGACGACGACAGACC  AGGGTGAAACTGGAGCAAAT  CGGATGATGAGGAATGGTCTAC  TTCTTTCTTCCTCCACACGCCT  TTCAACAGCTTCCCCAGGCA  AGACGGGGACGGAGACTTTC  CGTGAGCGAGGAGAATGCCA  GAGACAGGGAAAGAGGGAGAGGC  GCGGAATCAACTACCAGCCT | GGGCTTCCACTTGTTCTTCT  GATAGGCCCCCACAGTCGTC  CCGAGATTATCAGCATGACTCTTGA  AGCTGTTATTGCGGTTGTCACC  TTGCTCTCCAGCATGTTCTC  GCCGATGCTCTCGTTCTC  TTGTGATAAATGCCCCAACCAGG  TGCCTTGGAGGCAATGGTGT  CCCGAGATTATCAGCATGACTC  TGTGCATCGGAGTTGGATAC  CCAAGGTCATGCCTTTAGGGT  CGTTCTTGCGCAGCTGGAAT  TATCTCGGTGCACGTCATGG  AGACCATAAGAGCAGAGCGGA  AATGGGCTTGTGGATGACTGCC  TCAAACTCAGCACCGACCTC |

**Table S2.** Ramachandran plot statistical features of differentially regulated proteins in maize genotypes (Gurez local and GM6).

| **Gurez local** | **Protein identified** | **Ramachandran plot statistics (%)** | | | |
| --- | --- | --- | --- | --- | --- |
|  |  | **MFR**^a^ | **AAR**^b^ | **GAR**^c^ | **DR**^d^ |
|  | RNA-metabolising metallo-beta-lactamase family protein [Zea mays] | 65.9 | 21.8 | 5.2 | 7.1 |
|  | rough sheath1 [Zea mays] | 73.7 | 16.2 | 3.6 | 6.6 |
|  | Threonine dehydratase biosynthetic chloroplastic [Zea mays] | 76.4 | 16.9 | 2.8 | 3.9 |
|  | Thylakoidal processing peptidase 1 chloroplastic [Zea mays] | 66.9 | 22.9 | 3.8 | 6.4 |
|  | farnesylated protein 3 [Zea mays] | 62.7 | 13.3 | 12.0 | 12.0 |
|  | NHL domain-containing protein [Zea mays] | 64.6 | 25.3 | 4.5 | 5.6 |
|  | Aquaporin PIP2-1 [Zea mays | 81.4 | 13.6 | 2.9 | 2.1 |
|  | Auxin response factor 12 [Zea mays] | 62.9 | 24.6 | 5.7 | 6.8 |
|  | IND1(iron-sulfur protein required for NADH dehydrogenase)-like [Zea mays] | 81.9 | 14.0 | 2.3 | 1.8 |
|  | Splicing factor 3B subunit 5/RDS3 complex subunit 10 [Zea mays] | 90.0 | 10.0 | 0.0 | 0.0 |
|  | GINS complex protein [Zea mays] | 86.0 | 12.4 | 1.1 | 0.6 |
|  | F10K1.23 [Zea mays] | 75.1 | 17.1 | 3.9 | 3.9 |
|  | Translation initiation factor IF-2 [Zea mays] | 43.1 | 37.2 | 9.1 | 10.6 |
|  | Threonine ammonia-lyase OS=Zea mays | 73.6 | 18.9 | 3.9 | 3.5 |
|  | Rx_N domain-containing protein | 84.1 | 11.0 | 3.7 | 1.2 |
|  | Nodulin-like protein [Zea mays] | 84.5 | 9.1 | 3.6 | 2.7 |
|  | Peroxidase 2-like [Zea mays] | 77.4 | 15.7 | 2.5 | 4.4 |
|  | Tetratricopeptide repeat (TPR)-like superfamily protein [Zea mays] | 81.2 | 12.0 | 4.5 | 2.3 |
|  | Calcium-dependent protein kinase substrate protein [Zea mays] | 62.6 | 23.4 | 6.4 | 7.7 |
| **GM6** | LETM1-like protein[Zea mays] | 81.4 | 12.6 | 1.7 | 4.3 |
|  | Mitochondrial proton/calcium exchanger protein [Zea mays] | 77.8 | 14.7 | 3.0 | 4.5 |
|  | Flowering time control protein FCA-like [Zea mays] | 55.4 | 30.6 | 6.4 | 7.6 |
|  | RNA binding [Zea mays] | 75.7 | 19.3 | 3.6 | 1.4 |
|  | Mediator of RNA polymerase II transcription subunit 11 [Zea mays] | 51.8 | 31.0 | 8.2 | 9.0 |
|  | Glycosyltransferase family 28 C-terminal domain containing protein [Zea mays] | 82.8 | 13.5 | 2.9 | 0.8 |
|  | Nuclear pore complex protein NUP98A [Zea mays] | 73.1 | 20.5 | 5.2 | 1.3 |
|  | Importin subunit alpha-2, partial [Zea mays] | 70.8 | 17.9 | 5.7 | 5.7 |
|  | Aquaporin PIP2-1 [Zea mays] | 52.8 | 32.5 | 7.0 | 7.7 |
|  | Nuclear pore complex protein NUP155 [Zea mays] | 87.9 | 9.5 | 0.9 | 1.7 |

# Ramachandran plot statistics was performed using PHYRE2 Protein Fold Recognition Server (<http://www.sbg.bio.ic.ac.uk/phyre2/html/page.cgi?id=index>) and PDBsum (<https://www.ebi.ac.uk/thornton-srv/databases/cgi-bin/pdbsum/GetPage.pl?pdbcode=index.html>) online tools.

## a= Most favoured regions; b= Additional allowed regions; c= Generously allowed regions; d= Disallowed regions
